# Isolated Compounds from *Turpinia formosana* Nakai** Induce Ossification

**DOI:** 10.3390/ijms20133119

**Published:** 2019-06-26

**Authors:** Zuha Imtiyaz, Yi-Fang Wang, Yi-Tzu Lin, Hui-Kang Liu, Mei-Hsien Lee

**Affiliations:** 1 Clinical Drug Development of Herbal Medicine, Graduate Institute of Pharmacognosy, Taipei Medical University, Taipei 11031, Taiwan; 2 Graduate Institute of Pharmacognosy, College of Pharmacy, Taipei Medical University, Taipei 11031, Taiwan; 3 Division of Basic Chinese Medicine, National Research Institute of Chinese Medicine (NRICM), Ministry of Health and Welfare, Taipei 112, Taiwan; 4 Center for Reproductive Medicine & Sciences, Taipei Medical University Hospital, Taipei 11031, Taiwan

**Keywords:** bone formation, *Turpinia formosana*, 3,3′-di-*O*-methylellagic acid-4-*O*-α-l-arabinofuranoside, mineralization, alkaline phosphatase, estrogen receptors, osteoporosis

## Abstract

Bone metabolism is a homeostatic process, imbalance in which leads to the onset of diseases such as osteoporosis and osteopenia. Although several drugs are currently available to treat such conditions, they are associated with severe side effects and do not enhance bone formation. Thus, identifying alternative treatment strategies that focus on enhancing bone formation is essential. Herein, we explored the osteogenic potential of *Turpinia formosana* Nakai using human osteoblast (HOb) cells. The plant extract was subjected to various chromatographic techniques to obtain six compounds, including one new compound: 3,3′-di-*O*-methylellagic acid-4-*O*-α-l-arabinofuranoside (**1**). Compounds 3,3′-di-*O*-methylellagic acid-4-*O*-α-l-arabinofuranoside (**1**), gentisic acid 5-*O*-*β*-d-(6′-*O*-galloyl) glucopyranoside (**2**), strictinin (**3**), and (-)-epicatechin-3-*O*-*β*-d-allopyranoside (**6**) displayed no significant cytotoxicity toward HOb cells, and thus their effects on various osteogenic markers were analyzed. Results showed that **1**–**3** and **6** significantly increased alkaline phosphatase (ALP) activity up to 120.0, 121.3, 116.4, and 125.1%, respectively. Furthermore, **1**, **2**, and **6** also markedly enhanced the mineralization process with respective values of up to 136.4, 118.9, and 134.6%. In addition, the new compound, **1**, significantly increased expression levels of estrogen receptor-α (133.4%) and osteogenesis-related genes of Runt-related transcription factor 2 (Runx2), osteopontin (OPN), bone morphogenetic protein (BMP)-2, bone sialoprotein (BSP), type I collagen (Col-1), and brain-derived neurotropic factor (BDNF) by at least 1.5-fold. Our results demonstrated that compounds isolated from *T. formosana* possess robust osteogenic potential, with the new compound, **1,** also exhibiting the potential to enhance the bone formation process. We suggest that *T. formosana* and its isolated active compounds deserve further evaluation for development as anti-osteoporotic agents.

## 1. Introduction

Osteoporosis is a common systemic disorder characterized by defects in the microarchitecture of bone tissue, increased fragility of bones, and reduced bone mass [1]. Bone, being a metabolically active tissue, undergoes continuous remodeling throughout life. Bone remodeling primarily comprises two processes, namely, ossification and bone resorption, which are respectively orchestrated by osteoblasts and osteoclasts derived from different progenitor pools and hence are controlled by distinct molecular mechanisms [2,3]. Bone remodeling is important for maintaining bone mass, repairing micro-damage, preventing the excessive accumulation of old bone, and maintaining mineral homeostasis [4]. An imbalance in this metabolism causes disorders such as osteoporosis.

Several classes of drugs are available to treat osteoporosis, the most common of which are the bisphosphonates, an antiresorptive class of drugs whose mode of action targets osteoclasts. Although these drugs were found to be effective at controlling osteoporosis, their long-term use is associated with adverse effects particularly in patients with lower risk of fractures [5,6]. Furthermore, they are highly associated with osteonecrosis of the jaw and atypical femur fractures [7]. In addition to bisphosphonates, estrogen replacement therapies and vitamin D and calcium supplements are also widely used to manage osteoporosis. While the former is associated with severe side effects, including venous thromboembolic disorders, breast cancer, endometrial cancer, and cortical porosity in cases of selective estrogen receptor (ER) modulators [8,9,10], the efficacy of the latter is uncertain. Thus, identifying alternative therapeutic strategies to improve existing options is paramount [11,12,13].

Transcription factors, proteins, and hormones play vital roles in bone metabolism. Alkaline phosphatase (ALP) is a key component of bone metabolism and is expressed early in the development of bone and calcification of cartilage tissues, as well as on the cell surface of osteoblasts and in matrix vesicles. However, in later stages of differentiation, while expressions of other genes (e.g., osteocalcin) is upregulated, ALP expression declines [14]. Runt-related transcription factor 2 (Runx2) is an upstream transcription factor essential for osteoblast differentiation. Runx2 is a well-characterized transcriptional regulator that is expressed by chondrocytes and osteoblasts [15], and it regulates the expression of the collagen alpha-1 X chain (Col10a1) [16]. Collagen interacts with several other factors responsible for bone strength, thus contributing to the mechanical properties of bone [17,18,19]. Osteopontin (OPN) is also a key factor that binds to various extracellular molecules, including osteocalcin (OCN), fibronectin, and type I collagen (Col-1), thereby adding physical strength to the extracellular matrix. OPN also promotes the attachment of bone cells to the bone matrix [20,21]. Estrogen is an essential hormone associated with bone formation as it is involved in the growth and maturation of bone as well as in the regulation of bone turnover in adults. In addition, estrogen is required for the closure of the epiphyseal plate during bone growth [22], and its deficiency causes an increase in the number of the osteoclasts, leading to enhanced bone resorption [23,24].

Plant-derived compounds are an essential source of drug discovery and have been extensively explored against different disease conditions including osteoporosis. Members of the Staphyleaceae family of plants are rich in medicinal values [25,26], particularly those belonging to the *Turpinia* genus. *Turpinia ternate* showed cytotoxicity against *Artemia salina* larvae [27]. *T. pomifera* was found to possess antimalarial activity against *Plasmodium falciparum* [25]. Roots of *T. formosana* are used in traditional Taiwanese medicine to reduce swelling and pain and unclog the blood circulation [28]. The plant has been documented to possess numerous medicinal activities including antiviral activity against influenza [29,30]. *T. formosana* is also used in traditional formula preparations for ankylosing spondylitis and cervical spondylosis [31]. Considering the numerous medicinal properties of the plant including its bone-related activities, we herein explored the osteogenic effect of *T. formosana* using human osteoblast (HOb) cells.

## 2. Results

### 2.1. Isolated Compounds from Turpinia formosana

**Figure 1:**
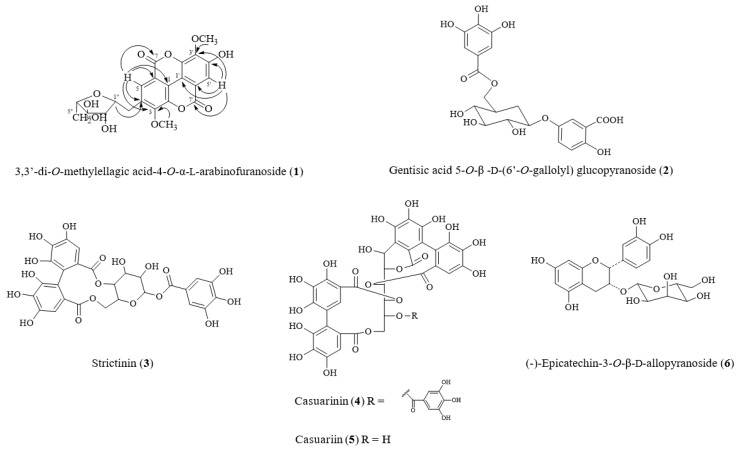
Structure of compounds isolated from *T. formosana.*

Column chromatography and semipreparative high-performance liquid chromatography (HPLC) were used for isolation and purification of compounds from the ethyl acetate (EtOAc) and *n*-butanol (*n*-BuOH) layers, as depicted in Chart S1, Supplementary Materials. We identified six major compounds (see Scheme S1, Supplementary Materials). On the basis of physical and spectroscopic methods, we identified these compounds as 3,3′-di-*O*-methylellagic acid-4-*O*-α-l-arabinofuranoside (**1**) (Figure S1A–E, supplementary material), gentisic acid 5-*O*-*β*-d-(6′-*O*-galloyl) glucopyranoside (**2**) (Figure S2A–E, supplementary material) [32], strictinin (**3**) (Figure S3A) [33], casuarinin (**4**) (Figure S4A–E) [33], casuariin (**5**) (Figure S5A–E) [33], and (-)-epicatechin-3-*O*-*β*-d-allopyranoside (**6**) (Figure S6A–E) [34] (see Figure 1). 3,3′-Di-*O*-methylellagic acid-4-*O*-α-l-arabinofuranoside (**1**) is a new compound, being isolated and reported for the first time in our study.

### 2.2. Structural Elucidation of New Compound 1 from T. formosana

Compound **1** was obtained as a yellow amorphous powder and was isolated from the EtOAc layer of the *T. formosana* extract. Its molecular formula was established to be C_21_H_18_O_12_ based on the prominent pseudomolecular ion peaks at *m/z* 461.1 [M–H]^–^ in electrospray ionization-mass spectroscopy (ESI-MS) and 461.0719 [M–H]^–^ in high-resolution mass spectroscopy (HRMS). The UV spectrum of **1** exhibited absorption maxima in methanol (MeOH) at 211, 246, and 367 nm, which were possibly identified as carboxyl and phenyl groups, and benzene rings. The ^1^H and ^13^C nuclear magnetic resonance (NMR) spectra (DMSO-*d*_6_) of **1** showed signals assignable to two non-equivalent methoxyl groups [δ_H_ 4.03 (3H, *s*); δ_C_ 61.4 (3-OCH_3_) and δ_H_ 4.07 (3H, *s*); δ_C_ 61.0 (3′-OCH_3_)] and two aromatic protons [δ_H_ 7.45 and δ_H_ 7.70 (each 1H, *s*)]. Compound **1** showed signals ascribable to a methylellagic acid moiety with seven pairs of quaternary carbons signals [δ_C_ 113.8, 110.8 (C-1, 1′); δ_C_ 141.4, 140.8 (C-2, 2′); δ_C_ 141.9, 140.2 (C-3, 3′); δ_C_ 150.7, 152.9 (C-4, 4′); δ_C_ 111.6, 111.7 (C-5, 5′); δ_C_ 111.6, 112.5 (C-6, 6′); δ_C_ 158.4, 158.2 (C-7, 7′)] [27,35]. The moiety was also supported by the heteronuclear multiple-bond correlations (HMBCs) from H-5 (δ 7.70, 1H, *s*) to C-1, -3, -4, -6, and -7 as well as H-5′ (δ 7.45, 1H, *s*) to C-1′, -3′, -4′, -6′, and -7′. In addition, two-dimensional NMR spectroscopic data allowed the identification of sugar protons. Correlation spectroscopy (COSY) was used to determine the connectivity around the sugar from H-1′ to H-6′ [δ_H_ 5.63/4.23, δ_H_ 4.23/3.87, δ_H_ 3.87/3.97, δ_H_ 3.97/3.50, δ_H_ 3.97/3.62]. Comparing with acid hydrolysis, the sugar was identified by HPLC analysis of **1** as α-l-arabinose [36]. Long-range correlations seen in HMBC data were observed between 3-OCH_3_ (δ_H_ 4.03, 3H, *s*) and C-3 (δ_C_ 141.9); 3′-OCH_3_ (δ_H_ 4.07, 3H, *s*) and C-3′ (δ_C_ 140.2); and H-1″ (δ_H_ 5.63) and C-4 (δ_C_ 150.7). This showed the connectivity of the methoxy and arabinofuranosyl moieties in **1**. Accordingly, **1** was characterized as the new ellagic acid derivative 3,3′-di-*O*-methylellagic acid-4-*O*-α-l-arabinofuranoside (Table 1).

### 2.3. Cytotoxicity of Compounds Isolated from T. formosana toward HOb Cells

**Figure 2:**
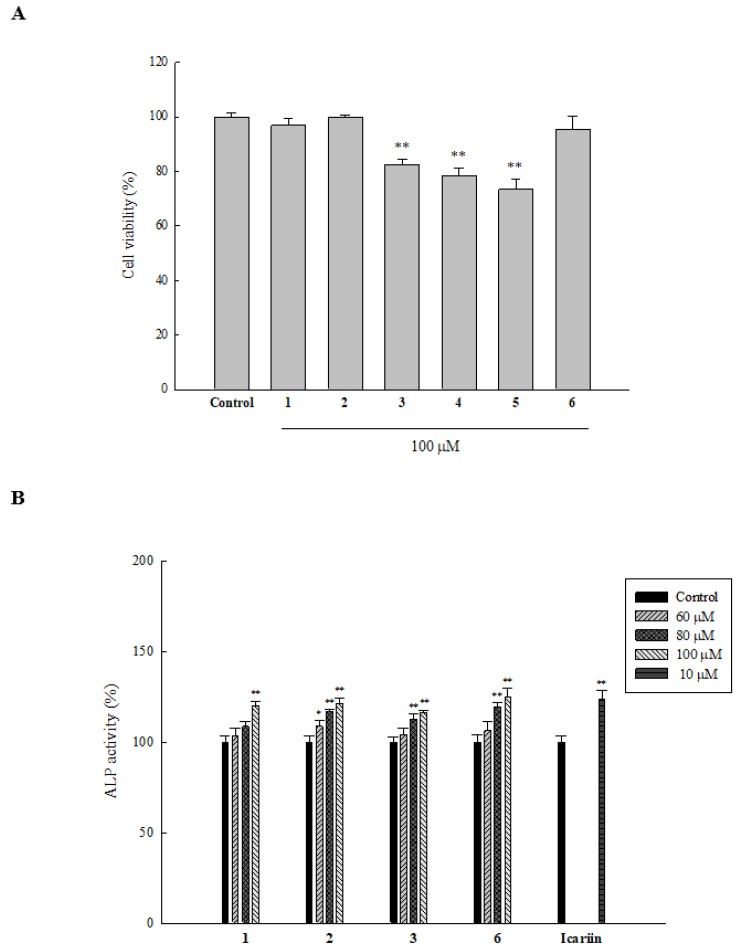
Cytotoxicity and induction ALP activity by isolated compounds in HOb cells. Cells were seeded in a 96-well plate, and after 24 h, 100 µM samples were added. (**A**) After five days, an MTT assay was performed to analyze cell viability. (**B**) After three days, ALP activity was detected by performing an ALP assay using bicinchoninic acid (BCA) protein to normalize protein expression of cells. Data are expressed as the mean ± SD, * *p* ≤ 0.05, ** *p* ≤ 0.01 compared to the control, and all experiments were performed in triplicate.

HOb cells were treated with the isolated compounds (100 µM) for three days, before analyzing cell viability using 3-(4,5-dimethylthiazol-2-yl)-2,5-diphenyltetrazolium bromide MTT assay. Results showed that cell viability of groups treated with **1**, **2**, **3**, and **6** were all above 80%, and hence, we studied the osteogenic activities of those compounds (see Figure 2A).

### 2.4. Effect of Isolated Compounds on ALP Activity in HOb Cells

ALP is one of the early biomarkers of bone formation, and studies have shown that it plays a vital role in mineral deposition. Hence, we considered it a foundational biomarker like many other research groups. On treating HOb cells with 100 µM of **1**, **2**, **3**, and **6** for three days, an increase in the ALP activity was observed (data not shown). Following this observation, we next examined whether the compounds could increase ALP activity in a concentration-dependent manner. As depicted in Figure 2B, **1** and **2** exhibited a concentration-dependent increase in ALP activity with 60, 80, and 100 µM increasing ALP activity up to 103.7, 108.8, and 120.0% for **1** and 108.9, 117.0, and 121.3% for **2**, respectively. However, **3** and **6** only displayed a significant increase in ALP activity at concentrations of 80 and 100 µM. Specifically, **3** at 80 and 100 µM increased ALP activity to 112.8 and 116.4%, whereas the same concentrations for **6** increased ALP activity to 119.7 and 125.1%, respectively (* *p* ≤ 0.05, ** *p* ≤ 0.01 compared to the control).

### 2.5. Effect of Isolated Compounds on Mineralization in HOb Cells

**Figure 3:**
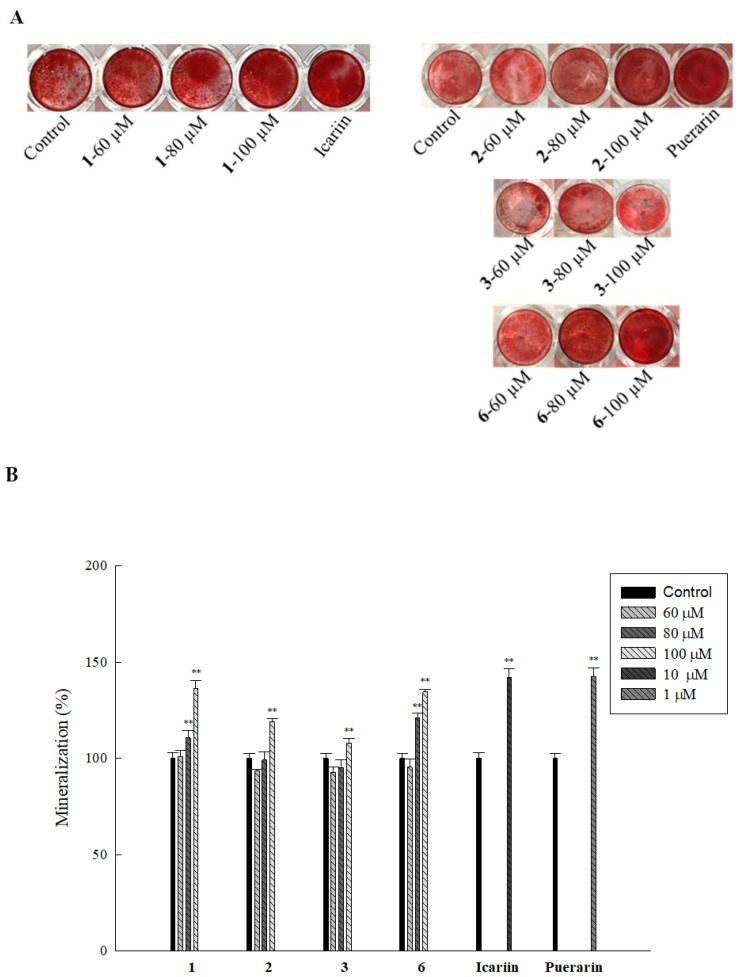
Isolated compounds increase mineralization in HOb cells. Cells were seeded in a 48-well plate for three days, after which fresh osteoblast differentiation media (ODM) miner containing inducers and samples were added. Subsequently, every two days, fresh ODM miner, inducers, and samples were added until the 11th day. Mineralization was then detected by performing an alizarin red assay. (**A**) Pictures show mineral deposition after addition of the alizarin red dye, and (**B**) the graph shows quantitative data obtained after adding 10% cetylpyridinium chloride as a de-stain to dissolve the crystals. Data are expressed as the mean ± SD (** *p* ≤ 0.01 compared to the control), and all experiments were performed in triplicate.

Extracellular matrix mineralization is an essential step in bone formation, which is controlled by several genetic pathways which regulate the homeostasis of minerals required for bone mineral formation [37]. On performing an alizarin red assay to determine mineralization by the active compounds at various concentrations, we found that while **1** and **6** at 80 and 100 µM increased mineralization up to 110.8%, 136.4% for **1** and 120.8%, and 134.6% for **6**, significant increase in mineralization was only observed at 100 µM for **2** (118.9%) and **3** (107.9 %) (Figure 3A,B). These results suggest that compared to **2** and **3**, **1** and **6** are more effective at increasing mineral deposition in HOb cells.

### 2.6. Effect of Isolated Compounds on ER Expression in HOb Cells

**Figure 4:**
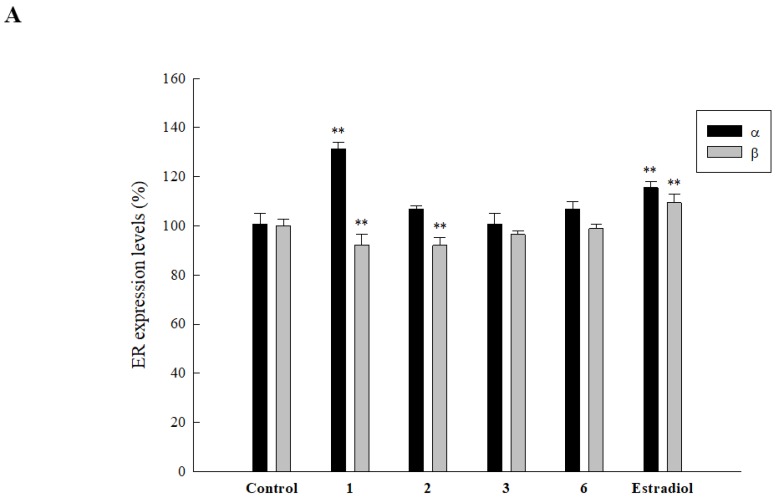

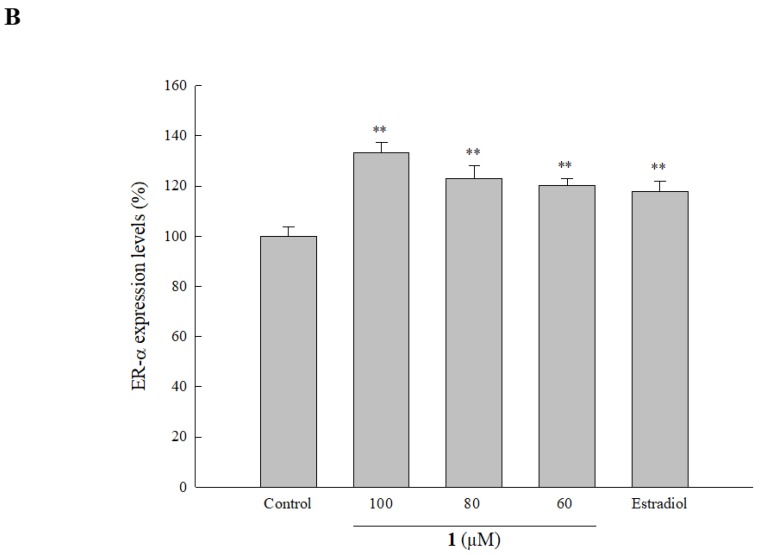
Effect of isolated compounds on ER expression in HOb cells. Cells were seeded in a 96-well plate and after 24 h, 100 µM of samples and 1 µM estradiol (positive control) were added. After five days, (**A**) ER-α/β expression levels were detected. (**B**) The dose-dependent effect of **1** on the expression of ER-α was analyzed. Data are expressed as the mean ± SD (** *p* ≤ 0.01 compared to the control), and all experiments were performed in triplicate.

Studies showed the significance of ERs and their involvement in bone formation. In order to examine whether any of the active compounds modulate expression levels of ERs, we analyzed the effect of **1**, **2**, **3**, and **6** on ER-α in HOb cells. Results showed that only **1** significantly increased protein expression of ER-α (see Figure 4A). Further analysis demonstrated that **1** increased ER-α expression in a concentration-dependent manner, with 60, 80, and 100 µM of the compound increasing ER-α expression to 120.3, 123.2, and 133.4%, respectively (see Figure 4B). This indicates that **1** modulates the expression of ER-α, thus activating the related pathway. Furthermore, we also examined the effect of the compounds on ER-β expression as it is believed to function as an antagonist to ER-α. We found that whereas **3** and **6** had no significant effect on ER-β, **1** and **2** at 100 µM significantly decreased the expression level of ER-β up to 92.2 and 92.2%, respectively (see Figure 4A) (** *p* ≤ 0.01 compared to the control).

### 2.7. Effect of Isolated Compounds on Genetic Markers of Bone Formation in HOb Cells

**Figure 5:**
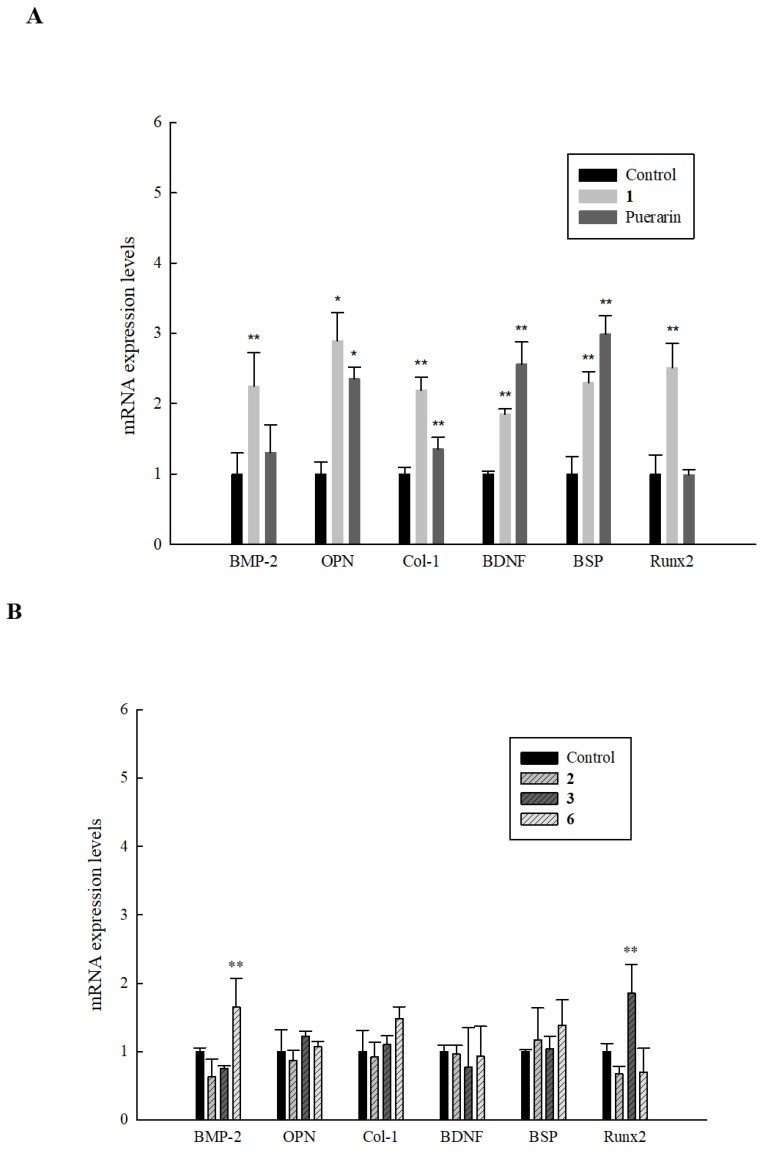
Effect of active compounds on bone formation-related genes in HOb cells. Cells were seeded in 6-cm dishes and after 24 h, fresh ODM containing (**A**) 100 µM of **1** and 1 µM puerarin (the positive control)**,** or (**B**) 100 µM of **2**, **3**, and **6** was added to cells. This was followed by mRNA isolation and reverse transcription. Expression levels were detected by performing a real-time PCR. Data are expressed as the mean ± SD (* *p* ≤ 0.05, ** *p* ≤ 0.01 compared to the control), and all experiments were performed in triplicate.

Bone metabolism is a complex process involving various pathways which interact to regulate bone formation. We tested the effect of all active compounds on the expression of certain key genes and transcription factors involved in osteoblastogenesis and osteoclast apoptosis in HOb cells. After treating cells with **1** for 24 h, we observed a significant increase in the mRNA expression of all the tested genes, with the expression in OPN (2.9-fold) being the highest. Expression levels of bone morphogenetic protein (BMP)-2, brain-derived neurotropic factor (BDNF), Runx2, Col-1, and bone sialoprotein (BSP) were increased by 2.2-, 1.9-, 2.5-, 2.2-, and 2.3-fold respectively (see Figure 5A). Expression levels were higher compared to puerarin (the positive control) for all the markers except for BDNF and BSP. On analyzing the effect of **2**, **3**, and **6** on the same selected biomarkers, results showed that **3** increased the expression of Runx2 by 1.8-fold and **6** increased the expression of BMP-2 to 1.7-fold, Figure 5B, * *p* ≤ 0.05, ** *p* ≤ 0.01 compared to the control. These results demonstrated that in contrast to other active compounds, **1** had a significant effect on all of the selected biomarkers, thereby identifying it as a potent osteogenic inducer*.*

## 3. Discussion

Nature-derived compounds serve as an essential source of drug discovery. An increasing body of evidence has shown the osteogenic potential of plants and their active compounds. *Epimedium koreanum* and *E. sagittatum* from the Berberidaceae family contain epimedium flavonoids such as icariin, epimedin A, and epimedin C [38,39]. These active flavonoids are the main constituents with anti-osteoporotic activities of these *Epimedium* plants [40]. *Glycine max* from the Fabaceae family, commonly known as soybean, is rich in proteins and flavonoids like genistein and diadzien, which are categorized as phytoestrogens [41]. These phytoestrogens displayed significant effects on bone metabolism in post-menopausal women [42,43,44]. *Elaeis guineensis* from the Arecaceae family has a high content of vitamin E, which possesses anti-inflammatory and antioxidant properties, making it suitable for anti-osteoporotic activity [45,46]. A study showed that compounds isolated from *T. formosana* possessed anticancer and antioxidant activities [28]. Our findings, therefore, also add *T. formosana* as an important bone-formation material source to the growing list of natural bioactive agents identified to have activities against osteoporosis, which may be useful for developing osteoporosis treatment strategies.

Our data provides evidence that *T. formosana* possesses an osteogenic effect. This was indicated by increases in ALP activity, mineral deposition, ER-α expression, and expression levels of molecular markers (Runx2, BDNF, BSP, Col-1, BMP-2, and OPN) of bone formation by the isolated active compounds, suggesting that the active compounds mediated ossification activity. Out of the six isolated compounds, 3,3′-di-*O*-methylellagic acid-4-*O*-α-l-arabinofuranoside (**1**) is a novel compound, which was isolated and reported for the first time in our study. Compound **1** is an ellagic acid derivative, and is similar to a compound isolated from *T. ternata* [27]. Ellagic acid is a polyphenol that increases both ALP and osteocalcin (OCN) expression in male rats after tooth extraction [47]. Interestingly, several plant-derived compounds were previously shown to promote osteogenesis via regulating ER-α expression, which is essential for increases in both cortical and trabecular bone mass. ER-β suppresses the signaling response of ER-α that is involved in bone formation [48,49]. Concordant with those findings, we also discovered that **1** robustly upregulated ER-α expression and downregulated ER-β expression, suggesting that **1** may exert its osteogenic effects by modulating ER-α expression. Further in-depth mechanistic studies are needed to clarify exactly how **1** promotes osteogenesis [47].

Oxidative stress is caused by the overproduction of reactive oxygen species (ROS). Clinical studies have shown that ROS are associated with bone loss and low bone mineral density [50,51]. Oxidative stress damages fibronectin, a major component of extracellular matrix in bone, thus inhibiting osteoblastic mineralization [52,53]; it also activates pre-osteoclast differentiation and osteoblast apoptosis, thus enhancing bone resorption [54,55,56,57]. Given that antioxidant activity correlates with an increase in ALP [58] and that **2**, **3**, and **6** increased ALP activity, it is possible that upregulation of ALP activity by these compounds is due to their antioxidant activity. Interestingly, ALP was suggested to increase the concentration of inorganic phosphate, an enhancer of mineral formation, and to attenuate levels of extracellular pyrophosphate, an inhibitor of mineralization [59]. Concordant with those findings, **2**, **3**, and **6** elevated levels of mineral deposition. In our study, we found a similar effect, but we did not explore its antioxidant effect. Instead, we focused on studying its effect on enhancing bone formation, because the bone-related activities of **2** and **3** had never been reported before, making our study the first to describe their involvement in bone metabolism. Previous studies reported that **3** possesses antioxidant activity [60,61], which could partially explain why it increased ALP activity and mineral deposition. A recent study showed that (-)-epicatechin-3-*O*-*β*-d-allopyranoside (**6**) is a dominant compound in the ethanolic extract of *Davallia formosana*,** a plant with promising anti-osteoporotic activity. The anti-osteoporotic activity of **6** is mediated by inhibition of osteoclastogenesis [62]. However, in our study, as explained above, we focused on the bone formation-related activity of **6**. Compound **6** increased both ALP activity and mineralization, without displaying any significant effect on the molecular markers of bone formation except for BMP-2. This observation combined with results of previous studies suggest that **6** might be more effective in inhibiting bone resorption than promoting bone formation.

Bone metabolism is a dynamic process that is controlled by several signaling pathways. In particular, osteoblast differentiation is mainly controlled by BMP, fibroblast growth factor (FGF), and transcription factors such as Runx2. Runx2 is essential for regulating osteogenesis and also plays key roles in osteoblast maturation. In our study, we observed that **1** increased Runx2 expression up to 2.5-fold, suggesting that the compound enhances osteoblast maturation. Extracellular matrix in bone is predominantly composed of Col-1 protein which initiates build-up of the bone matrix. Our results showed that **1** increased Col-1 expression up to 2.2-fold, which is essential in providing strength and elasticity to the bone and the capacity for the deposition of other matrix components [63]. Upregulation of Runx2, Col-1, and ALP indicated the induction of osteogenesis by emodin in bone marrow mesenchymal stem cells (BMSCs) [64]. Intriguingly, **1** significantly increased the expression of OPN, which regulates matrix deposition. It was also found to increase expression levels of BDNF which is reported to mediate bone morphogenetic protein (BMP)-2 expression [65]. Expression levels of BMP-2 were also elevated by **1**, thus regulating the expression of Runx2 and increasing endochondral ossification and calcium deposition [66,67]. Our findings that **1** increased expression of all the essential genes involved in osteogenesis in HOb cells pinpoint **1** as an important natural product for enhancing bone formation.

In summary, our results suggested that compounds isolated from *T. formosana* can elevate ALP activity and mineralization. Additionally, the new compound 3,3′-di-*O*-methylellagic acid-4-*O*-α-l-arabinofuranoside (**1**) upregulated factors such as BMP-2, BSP, OCN, Runx2, Col-1, and BDNF that are responsible for bone formation, suggesting that **1** possesses ossification potential. Thus, the compound can possibly be used as a nutritional supplement or as a drug to enhance bone formation in the future.

## 4. Materials and Methods

### 4.1. Materials and Reagents

Alizarin red S, 3-(4,5-dimethylthiazol-2-yl)-2,5-diphenyltetrazolium bromide (MTT), dimethyl sulfoxide-*d*_6_ (DMSO-*d*_6_), *β*-glycerophosphate (*β*-GP), and methanol-*d*_4_ (Sigma-Aldrich, St. Louis, MO, USA). Cetylpyridinium chloride, *p*-nitrophenyl phosphate, sodium bicarbonate, and sodium phosphate (Mallinckrodt, St. Louis, MO, USA). Dulbecco′s phosphate-buffered saline (PBS) (Gibco, Burlington, ON, Canada). Dimethyl sulfoxide (DMSO), and Triton-X 100 (J-T Baker, Phillipsburg, NJ, USA). ACS-grade ethanol and methanol (MeOH) Echo chemical, Miaoli, Taiwan). *p*-Nitrophenyl phosphate, sodium bicarbonate, and paraformaldehyde (Alfa Aesar, Ward Hill, MA, USA).

### 4.2. General Experimental Techniques

HPLC analysis was performed using a Hitachi L-7100 pump and an L-7420 UV-VIS detector with a reversed-phase column (Biosil ODS-W, 4.6 × 250 mm, 10 × 250 mm; Biotic Chemicals, Taipei, Taiwan). UV spectra were recorded on a UV-1601 spectrophotometer (Shimadzu, Tokyo, Japan). ^1^H-NMR and ^13^C-NMR spectra were recorded on a Bruker DRX-500 MHz (Billerica, MA, USA) (^1^H at 500 MHz; ^13^C at 125 MHz), and chemical shifts are given in δ (ppm). Two-dimensional spectra were obtained through COSY, HMQC, and HMBC experiments. ESI-MS and HRMS were carried out using an LCQ mass spectrometer and Q Exactive™ Plus Hybrid Quadrupole-Orbitrap™ Mass Spectrometer (Thermo Finnegan, Waltham, MA, USA). All solvents were distilled before use. Solvents were removed from extracts by rotary evaporation N-1000 (Eyela, Tokyo, Japan) under reduced pressure at temperatures of up to 40 °C.

### 4.3. Plant Extract Preparation

Leaves of *T. formosana* were obtained from and identified by Dr. Ih-Sheng Chen, College of Pharmacy, Kaohsiung Medical University (Kaohsiung, Taiwan). An herbarium voucher specimen (M387) was deposited in the Graduate Institute of Pharmacognosy, Taipei Medical University (Taipei, Taiwan). Dried leaves (5.3 kg) were extracted using 95% ethanol (EtOH; 40 L, thrice). After filtration, the mixture was concentrated using a rotary evaporator (Eyela, Tokyo, Japan) and lyophilized with a freeze-dryer (Eyela, Tokyo, Japan), and 232.0 g of extract was obtained.

### 4.4. Isolation and Purification of Active Compounds

The ethanolic extract of *T. formosana* (TF) was suspended in an aqueous solution and partitioned using *n*-hexane, ethyl acetate (EtOAc), and *n*-butanol (*n*-BuOH), to obtain four layers: n-hexane at 87 g; EtOAc at 25.1 g; n-butanol at 41.8 g; and an aqueous solution at 56.2 g. The EtOAc and *n*-BuOH fractions were further subjected to chromatography using a Sephadex LH-20 (18–111 μm, Amersham Pharmacia Biotech, Stockholm, Sweden) and ethanol to methanol as the gradient mobile phase. This produced nine fractions from each layer (TFEA-1–9) and (TFBU-1–9), respectively. TFEA-3 was subjected to a C18 column gradient of H_2_O to MeOH to obtain 10 sub-fractions (TFEA-3-1–10); following this, TFEA-3-6 was eluted using a C18 gradient to obtain two sub-fractions (TFEA-3-6-1 and TFEA-3-6-2). CP-1 (109.7 mg) was collected after purification of TFEA-3-6-1 using HPLC RP-ODS 35% MeOH (see Chart S1, Supplementary Materials).

### 4.5. Cell Culture

Primary human osteoblast (HOb) cells from a normal femur of a 63-year-old Caucasian female were purchased from Cell Applications (San Diego, CA, USA). Cells were cultured in osteoblast growth medium (OGM, Cell Applications, San Diego, CA, USA) for proliferation. For differentiation, osteoblast differentiation medium (ODM, Cell Applications, San Diego, CA, USA) was used. ODM containing ascorbic acid, dexamethasone, and *β*-glycerophosphate was purchased from Sigma-Aldrich (St. Louis, MO, USA), for mineralization at 37 °C in a 5% CO_2_ humidified atmosphere [68] (ODM for mineralization is represented as ODM miner in this study).

### 4.6. MTT Assay for Cell Viability Assay

The viability of HOb cells was evaluated using an MTT assay. Cells at 4 × 10^3^ per well in OGM were seeded in a 96-well plate. After one day, fresh OGM with **1**–**6** was added to cells. After five days, medium was replaced by fresh OGM containing 0.5 mg/mL MTT reagent and incubated for 4 h at 37 °C in a 5% CO_2_ atmosphere. Medium was removed, and DMSO was used to dissolve the formazan crystals. The optical density (OD) was measured using a microplate reader (Bio-Tek Instruments, Winooski, VT, USA) at 600 nm [68,69].

### 4.7. ALP Activity Assay

Cells (4 × 10^3^ per well in OGM) were seeded in a 96-well plate. After one day, fresh OGM containing samples or positive controls was added. Three days after this, medium was removed, PBS was used for washing, and lysis buffer (0.1% Triton X-100 in PBS) was used to lyse cells. The lysate was separated into different wells for ALP and bicinchonic acid (BCA). The *p*-nitrophenyl phosphate substrate (in 6 mM NaHCO_3_-Na_2_CO_3_ buffer, pH 10) was added to wells with lysate for ALP and the BCA protein assay reagent to respective wells. The reaction was allowed to proceed for 1 h at 37 °C. ODs were measured using a microplate reader at 405 and 560 nm for ALP and BCA, respectively. ALP activity was determined by ALP value/BCA value, and normalized to the control as 100% [68,70].

### 4.8. Mineralization Assay

HOb cells were seeded at the density of 4 × 10^4^ cells per well in a 48-well plate using OGM. After three days, fresh ODM miner containing inducers, active compounds, or the positive control was added. Every two days, freshly prepared ODM miner containing test samples was added. After 11 days, medium was removed, PBS was used to wash HOb cells, and then 4% paraformaldehyde was added for 20 min at room temperature to fix the cells. Cells were washed with PBS twice, and 40 mM alizarin red S dye was added for 20 min at room temperature. After removing the dye, water was used to wash out any extra dye; this was followed by photographing the samples. For the quantitative analysis, 10% cetylpyridinium chloride (in 10 mM sodium phosphate, pH 7.0) was used to dissolve crystals, and the solution was transferred to a 96-well plate. A microplate reader was used to measure the OD at 550 nm [68,71].

### 4.9. RNA Isolation and Reverse Transcription

Gene expression levels in HOb cells were measured using a real-time polymerase chain reaction (PCR). HOb cells (7 × 10^5^) in OGM were seeded in 6-cm dishes. After one day, fresh ODM containing active compounds or the positive control was added. After 24 h, cells were collected and centrifuged for 5 min at 3000 rpm and 4 °C [68]. For RNA isolation, a High Pure RNA Isolation Kit (Roche, Mannheim, Germany) was used according to the manufacturer’s instructions. The concentration and quality of RNA samples were detected using a Nanodrop 2000c (Thermo Scientific, Waltham, MA, USA). The eluted RNA was stored at −80 °C for later analysis.

A high-capacity complementary (c)DNA reverse-transcription kit (Thermo Scientific, Waltham, MA, USA) was used to make cDNA according to the manufacturer’s instructions. The reaction took place in the following thermal cycle: 25 °C for 10 min, 37 °C for 120 min, 80 °C for 5 min, and 4 °C for 60 min. cDNA was stored at −20 °C until further analysis [72].

### 4.10. Real-Time Quantitative PCR Analysis

The real-time PCR mix contained 5 μL LightCycler^®^ 480 probe master (Roche, Mannheim, Germany), 0.4 μL forward and reverse primers, 0.2 μL Universal Probe Library (UPL) probes (Roche), and 3.4 μL nuclease-free H_2_O for each well. cDNA samples at 1 μL/well were added to a specific 96-well plate; this was followed by adding 9 μL of the reaction mix, to make a final volume of 10 μL in each well. Sequences of the primers and respective probe numbers are shown in Table 2. Gene expression results were analyzed using Livak’s formula (2^(−ΔΔCt)^ method), normalized against GAPDH expression (as a housekeeping gene) and are expressed as the relative expression compared to that of the control. Cycling parameters for the LightCycler^®^ 480 system (Roche) were set as follows: pre-incubation at 95 °C for 10 min, amplification followed by 50 cycles of 95 °C (for 10 s), 60 °C (for 30 s), and 72 °C (for 1 s), followed by a cooling step of 30 s at 40 °C [68].

### 4.11. Estrogen Receptor Expression Assay

HOb cells at a density of 4 × 10^3^ cells per well in OGM were seeded in a 96-well plate. After 24 h, fresh OGM containing active compounds or the positive control was added. After five days of incubation, expressions levels of ER-α and -β were analyzed using ESR1 and ESR2 (human) cell-based enzyme-linked immunosorbent assay (ELISA) kits (Abnova, Walnut, CA, USA) according to the manufacturer’s instructions [73].

### 4.12. Statistical Analysis

Statistical analyses were performed with SigmaPlot (11.0, SYSTAT, Chicago, IL, USA). A one-way analysis of variance (ANOVA) with the Student–Newman–Keuls analysis was conducted to determine statistical significance. *p* < 0.05 was considered statistically significant.

## Figures and Tables

**Table 1:** ^1^H-NMR and ^13^C-NMR spectral data of **1** (δ values, in DMSO-*d_6_*, *J* in Hz).

Position	δ_C_	δ_H_ (multi, *J* in Hz)	HMBC (H→C)
methylellagic acid			
1	113.8		
2	141.4		
3	141.9		
4	150.7		
5	111.6	7.70 (1H, *s*)	C-1, C-3, C-4, C-6, C-7
6	111.6		
7	158.4		
1′	110.8		
2′	140.8		
3′	140.2		
4′	152.9		
5′	111.7	7.45 (1H, *s*)	C-1′, C-3′, C-4′, C-6′, C-7′
6′	112.5		
7′	158.2		
3-OCH_3_	61.4	4.03 (3H, *s*)	
3′-OCH_3_	61.0	4.07 (3H, *s*)	
arabinose			
1″	107.5	5.63 (1H, *brs*)	C-4
2″	82.1	4.23 (1H, *d*, *J* = 3.9 Hz)	
3″	76.6	3.87 (1H, *m*)	
4″	86.1	3.97 (1H, *m*)	
5″	60.9	3.50 (1H, *m*) 3.62 (1H, *m*)	

J, coupling constant expressed in Hz; s, singlet; brs, broad singlet; d*,* doublet; m, multiplet; H, proton; ‘ and “, depicts carbon position*.*

**Table 2:** Primer and probe combination used for the real-time PCR.

		Sequence (5′→ 3′)	Probe Number
Runx-2	Forward Reverse	CAGTGACACCATGTCAGCAA GCTCACGTCGCTCATTTTG	41
OPN	Forward Reverse	GGGCTTGGTTGTCAGCAG TGCAATTCTCATGGTAGTGAGTTT	63
BMP-2	Forward Reverse	CGGACTGCGGTCTCCTAA GGAAGCAGCAACGCTAGAAG	49
BSP	Forward Reverse	GATTTCCAGTTCAGGGCAGT TCTCCTTCATTTGAAGTCTCCTCT	63
Col-1	Forward Reverse	AGGTCCCCCTGGAAAGAA AATCCTCGAGCACCCTGA	60
BDNF	Forward Reverse	AGAATCGGAACCACGATTTG TCTCACCTGGTGGAACTCG	70
GAPDH	Forward Reverse	AGCCACATCGCTCAGACAC GCCCAATACGACCAAATCC	60

## Supplementary Materials

Supplementary materials can be found at https://www.mdpi.com/1422-0067/20/13/3119/s1.
