# Supplementary material for: Isolated Compounds from Turpinia formosana Nakai Induce Ossification
Source: Int J Mol Sci. 2019 Jun 26;20(13):3119. doi: 10.3390/ijms20133119 (PMC6651545; doi:10.3390/ijms20133119)
Supplement: Supplementary file 1 [file ijms-20-03119-s001.pdf]

Isolated compounds from *Turpinia formosana* Nakai induce ossification

Zuha Imtiyaz[1], *Yi-Fang Wang*[2], *Yi-Tzu Lin*[1], *Hui-Kang Liu*[1,3], *Mei-Hsien Lee*[1,2,4*]

[1] PhD in Clinical drug development of herbal medicine, Graduate institute of Pharmacognosy, Taipei Medical University, Taipei city, 110, Taiwan

d339105004@tmu.edu.tw (Z.I); d339102003@tmu.edu.tw (Y.-T.L); hk.liu@nricm.edu.tw (H.-K.L); lmh@tmu.edu.tw (L.-M.H)

[2] Graduate Institute of Pharmacognosy, College of Pharmacy, Taipei Medical University, Taipei city, 110, Taiwan

m303099010@tmu.edu.tw (Y.-F.W)

[3]Division of Basic Chinese Medicine, National Research Institute of Chinese Medicine (NRICM), Ministry of Health and Welfare, Taipei city, 112, Taiwan

[4]Center for Reproductive Medicine & Sciences, Taipei Medical University Hospital, Taipei 110, Taiwan

Contents

Chart S1. Isolation and purification of compounds from *Turpinia formosana*

Scheme S1. Spectral data of isolated compounds

Figure S 1A. ^{1}H NMR (DMSO-d_6, 500 MHz) spectrum of **1**

Figure S 1B. ^{13}C NMR (DMSO-d_6, 125 MHz) spectrum of **1**

Figure S 1C. ^{1}H-^{1}H COSY spectrum of **1**

Figure S 1D. HSQC spectrum of **1**

Figure S 1E. HMBC spectrum of **1**

Figure S 2A. 1H NMR (Methanol-d4, 500 MHz) spectrum of **2**

Figure S 2B. 13C NMR (Methanol-d4, 125 MHz) spectrum of **2**

Figure S 2C. 1H-1H COSY spectrum of **2**

Figure S 2D. HSQC spectrum of **2**

Figure S 2E. HMBC spectrum of **2**

Figure S 3A. ^{1}H NMR (acetone-d_6, 500 MHz) spectrum of **3**

Figure S 4A. ^{1}H NMR (acetone-d_6, 500 MHz) spectrum of **4**

Figure S 4B. ^{13}C NMR (acetone-d_6, 125 MHz) spectrum of **4**

Figure S 4C. ^{1}H-^{1}H COSY spectrum of **4**

Figure S 4D. HSQC spectrum of **4**

Figure S 4E HMBC spectrum of **4**

Figure S 5A. ^{1}H NMR (acetone-d_6, 500 MHz) spectrum of **5**

Figure S 5B. ^{13}C NMR (acetone-d_6, 125 MHz) spectrum of **5**

Figure S 5C ^{1}H-^{1}H COSY spectrum of **5**

Figure S 5D. HSQC spectrum of **5**

Figure S 5E. HMBC spectrum of **5**

Figure S 6A. ^{1}H NMR (DMSO-d_6, 500 MHz) spectrum of **6**

Figure S 6B ^{13}C NMR (DMSO-d_6, 125 MHz) spectrum of **6**

Figure S 6C ^{1}H-^{1}H COSY spectrum of **6**

Figure S 6D. HSQC spectrum of **6**

Figure S 6E HMBC spectrum of **6**

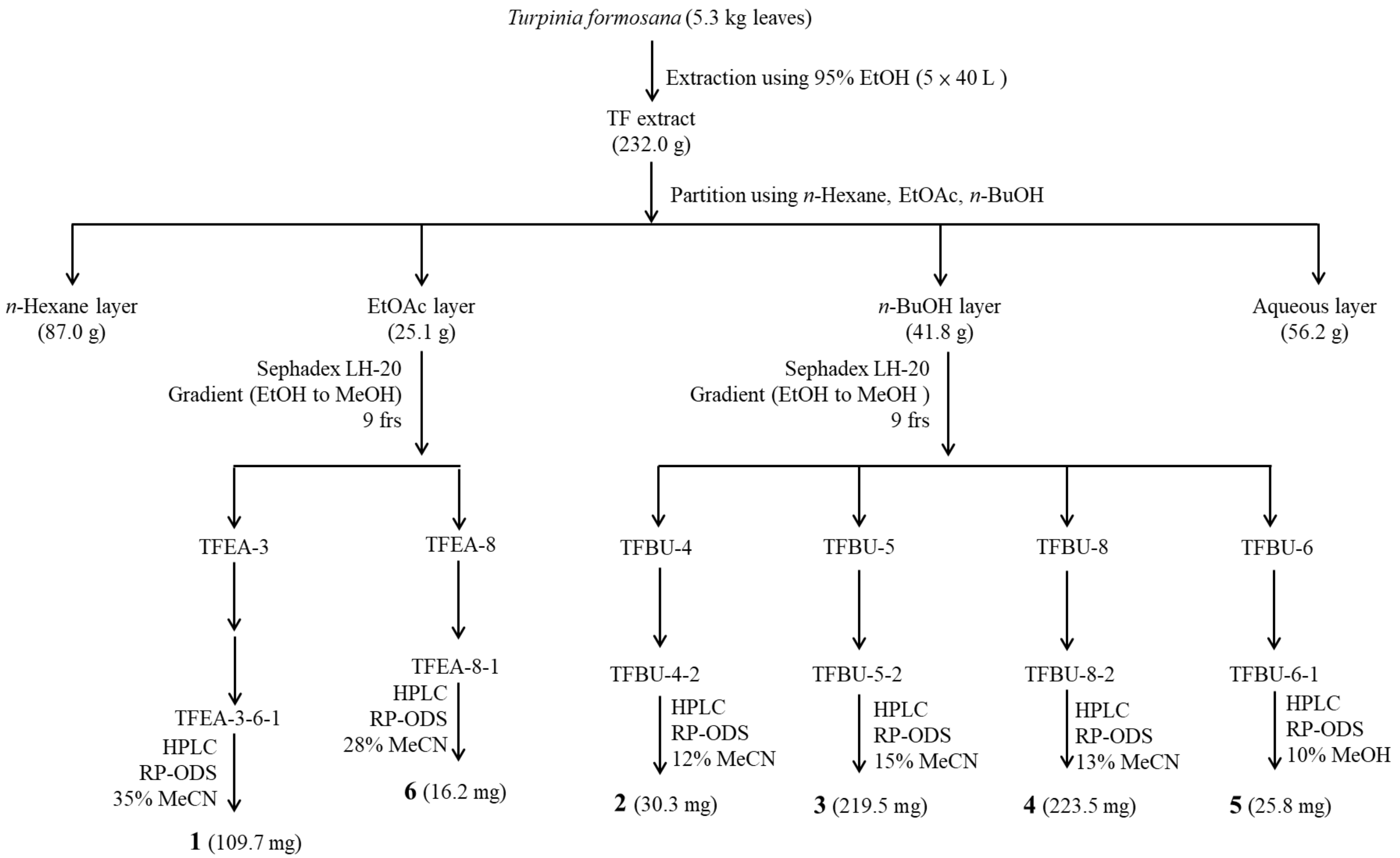

Chart S1. Isolation and purification of compounds from *Turpinia formosana*.

Scheme S1. Spectral data of isolated compounds

3,3′-Di-*O*-methylellagic acid-4-*O*-α-L-arabinofuranoside (**1**). Yellow powder, UV (MeOH) λ_{max} (log ε): 367 (4.13), 246 (4.74), 211 (5.15). ESI-MS (negative) m/z 461.1 [M – H]$^-$

^{1}H-NMR (500 MHz, DMSO-d_6)

δ: 7.70 (1H, *s*, H-5), 7.45 (1H, *s,* H-5′), 5.63 (1H, *brs*, H-1″), 4.23 (1H, *d*, *J*= 3.9 Hz, H-2″) , 4.07 (3H, *s*, 3′- OCH_3), 4.03 (3H, *s*, 3- OCH_3), 3.97 (1H, *m*, H-4″) , 3.87 (1H, *m*, H-3″), 3.62 (1H, *m*, H-5″), 3.50 (1H, *m*, H-5″)

^{13}C-NMR (125 MHz, DMSO-d_6)

δ: 158.4 (C-7), 158.2 (C-7′), 152.9 (C-4′), 150.7 (C-4), 141.9 (C-3), 141.4 (C-2), 140.8 (C-2′), 140.2 (C-3′), 113.8 (C-1), 112.5 (C-6′), 111.7 (C-5′), 111.6 (C-5), 111.6 (C-6), 110.8 (C-1′), 107.5 (C-1″), 86.1(C-4″), 82.1 (C-2″), 76.6 (C-3″), 61.4 (3- OCH_3), 61.0 (3′- OCH_3), 60.9 (C-5″)

Gentistic acid 5-*O*-*β*-D-(6′-*O*-galloyl) glucopyranoside (**2**). Brown powder, UV (MeOH) λ_{max} (log ε): 209 (5.09). ESI-MS (negative) m/z 467.2 [M – H]$^-$

^{1}H-NMR (500 MHz, methanol-d_4)

δ: 7.77 (1H, *d*, *J*= 3.1 Hz, H-6), 7.30 (2H, *s*, H-galloyl), 7.06 (1H, *dd*, *J*= 3.1, 8.9 Hz, H-4), 6.72 (1H, *d*, *J*= 8.9 Hz, H-3), 4.90 (1H, *d*, *J*= 7.6 Hz, H-1′), 4.75 (1H, *d*, *J*= 11.9 Hz, H-6′), 4.10 (1H, dd, J=8.6, 11.9 Hz, H-6′), 3.86 (1H, *t*, *J*=8.6 Hz, H-5′), 3.51 (1H, *t*, *J*=9.3 Hz, H-3′), 3.46 (1H, *t*, *J*=9.3 Hz, H-2′), 3.34 (1H, *d*, *J*=6.6 Hz, H-4′)

^{13}C-NMR (125 MHz, methanol-d_4)

δ: 176.0 (1-COOH), 168.7 (COO-), 158.0 (C-2), 151.3 (C-5), 146.6(C-3″, 5″), 139.8 (C-4″), 124.2 (C-4), 121.1 (C-1″), 119.7 (C-1) , 117.9 (C-3), 117.3 (C-6), 110.2 (C-2″, 6″), 103.2 (C-1′), 77.7 (C-3′), 75.9 (C-5′), 74.9 (C-2′), 71.9 (C-4′), 66.0 (C-6′)

Strictinin (**3**). Brown powder, UV (MeOH) λ_{max} (log ε): 272 (4.50), 211 (5.30). ESI-MS (negative) m/z 633.1 $[M – H]^{–}$

^{1}H-NMR (500 MHz, acetone-d_6)

δ: 7.17 (2H, *s*, H-galloyl), 6.69 (1H, *s*), 6.58 (1H, *s*), 5.70 (1H, *d*, *J* = 8.1 Hz, H-1), 5.18 (1H, *dd*, *J* = 13.3, 6.3 Hz, H-6), 4.87 (1H, *t*, *J* = 9.7 Hz, H-4), 4.08 (1H, *dd*, *J* = 9.7, 6.3 Hz, H-5), 3.80 (1H, *t*, *J* = 9.7 Hz, H-3), 3.74 (1H, *d*, *J* = 13.3 Hz, H-6), 3.68 (1H, *t*, *J* =8.7 Hz, H-2)

Casuarinin (**4**). Cameo brown powder, UV (MeOH) λ_{max} (log ε): 210 (5.45). ESI-MS (negative) m/z 935.0 $[M – H]^{–}$

^{1}H-NMR (500 MHz, acetone-d_6)

δ: 7.10 (2H, *s*, H-galloyl), 6.48, 6.54, 6.76 (each 1H, *s*, H-HHDP), 5.62 (1H, *dd*, *J* = 8.8, 3.0 Hz, H-1), 5.40 (1H, *d*, *J*= 5.0 Hz, H-4), 5.39 (1H, *brs*, H-3), 5.32 (1H, *m*, H-5), 4.78 (1H, *dd*, *J*= 13.5, 3.0 Hz, H-6), 4.68 (1H, *dd*, *J*= 5.0, 1.5 Hz, H-2), 4.06 (1H, *d*, *J*= 13.5 Hz, H-6)

^{13}C-NMR (125 MHz, acetone-d_6)

δ: 169.7, 168.8, 168.6, 164.0 (-COO^-),165.7 (galloyl-COO^-), 138.6, 136.6, 135.5, 134.6 (HHDP C-5), 146.3, 145.7, 145.2, 145.1, 144.7, 144.5, 143.9, 143.6 (HHDP C- 4,6), 145.9 (galloyl-3,5), 139.1 (galloyl-4), 121.2 (galloyl-1), 110.1 (galloyl-2,6), 116.9, 108.3, 107.0, 105.3 (HHDP C- 3), 127.7, 127.2, 125.1, 120.9 (HHDP C-2), 116.3, 116.0, 115.9, 115.2 (HHDP C-1), 76.7 (C-2), 74.3 (C-4), 71.2 (C-5), 69.9 (C-3), 67.7 (C-1), 64.6 (C-6)

Casuariin (**5**). Light brown powder, UV (MeOH) λ_{max} (log ε): 209 (5.30). ESI-MS (negative) m/z 783.1 $[M - H]^-$

^{1}H-NMR (500 MHz, acetone-d_6)

δ: 6.73, 6.52, 6.38 (each 1H, *s,* H-HHDP), 5.44 (1H, *t*, *J*= 2.5 Hz, H-3), 5.32 (1H, *d*, *J*= 5.0 Hz, H-1), 5.05 (1H, *dd*, *J*= 8.5, 2.5 Hz, H-4), 4.64 (2H, *m*), 4.08 (1H, *dd*, *J*=8.5, 2.5 Hz, H-5), 3.83 (1H, *d*, *J*=12.5 Hz)

^{13}C-NMR (125 MHz, acetone-d_6)

δ: 170.2, 169.6, 168.7, 165.5 (-COO^-), 146.0, 145.7, 145.1, 145.0 (HHDP C-4, 4′), 144.3, 144.2, 143.6, 143.5 (C-6, 6′), 138.7, 136.5, 135.6, 134.8 (C-5, 5′), 127.3, 127.0, 125.1, 119.8 (C-2, 2′), 116.9, 116.3, 116.1, 115.9 (C-1,1′), 114.9, 108.5, 106.9, 105.1 (C-3, 3′), 76.9, 76.8 (C-4), 70.6 (C-3), 68.1 (C-5), 68.0, 67.0 (C-1)

(-)-Epicatechin-3-*O*-*β*-D-allopyranoside (**6**). Light yellow powder, UV (MeOH) λ_{max} (log ε): 280 (3.68), 217 (4.32). ESI-MS (negative) m/z 451.1 $[M - H]^-$

^{1}H-NMR (500 MHz, DMSO-d_6)

δ: 9.19, 8.95, 8.69, 8.66 (each 1H, *s*, 5, 7, 3′ ,4′-OH), 6.89 (1H, *d*, *J*=2.0 Hz, H-2′), 6.68 (1H, *dd*, *J*=8.2, 2.0 Hz, H-6′), 6.60 (1H, *d*, *J*=8.2 Hz, H-5′), 5.88 (1H, *d*, *J*= 2.3 Hz, H-6), 5.74 (1H, *d*, *J*= 2.3 Hz, H-8), 5.13 (1H, *d*, *J*= 3.1 Hz, H-2), 4.75 (1H, *d*, *J*= 3.5 Hz, 3″-OH), 4.57 (2H, *dd*, *J*= 10.0, 6.9 Hz, 2″,4″-OH), 4.51 (1H, *d*, *J*= 7.5 Hz, H-1″), 4.35 (1H, *t*, *J*= 5.7 Hz, 6″-OH), 4.22 (1H, *m*, H-3), 3.79 (1H, *dd*, *J*=6.1, 3.0 Hz, H-3″), 3.65 (1H, *m*, H-6″), 3.49 (1H, *m*, H-5″), 3.40 (1H, *m*, H-6″), 3.25 (1H, *m*, H-4″), 3.11 (1H, *m*, H-2″), 2.68 (1H, *dd*, *J*= 16.0, 5.0 Hz, H-4), 2.33 (1H, *dd*, *J*= 16.0, 7.8 Hz, H-4)

^{13}C-NMR (125 MHz, DMSO-d_6)

δ: 156.6 (C-7), 156.2 (C-5), 155.1 (C-8a), 144.4 (C-4′), 144.2 (C-3′), 129.6 (C-1′), 118.6 (C-6′), 115.3 (C-2′), 114.7 (C-5′), 99.6 (C-1″), 98.5 (C-4a), 95.2 (C-8), 94.0 (C-6), 76.7 (C-2), 74.4 (C-5″), 72.4 (C-3), 71.5 (C-3″), 70.6 (C-2″), 67.7 (C-4″), 61.7 (C-6″), 23.0 (C-4

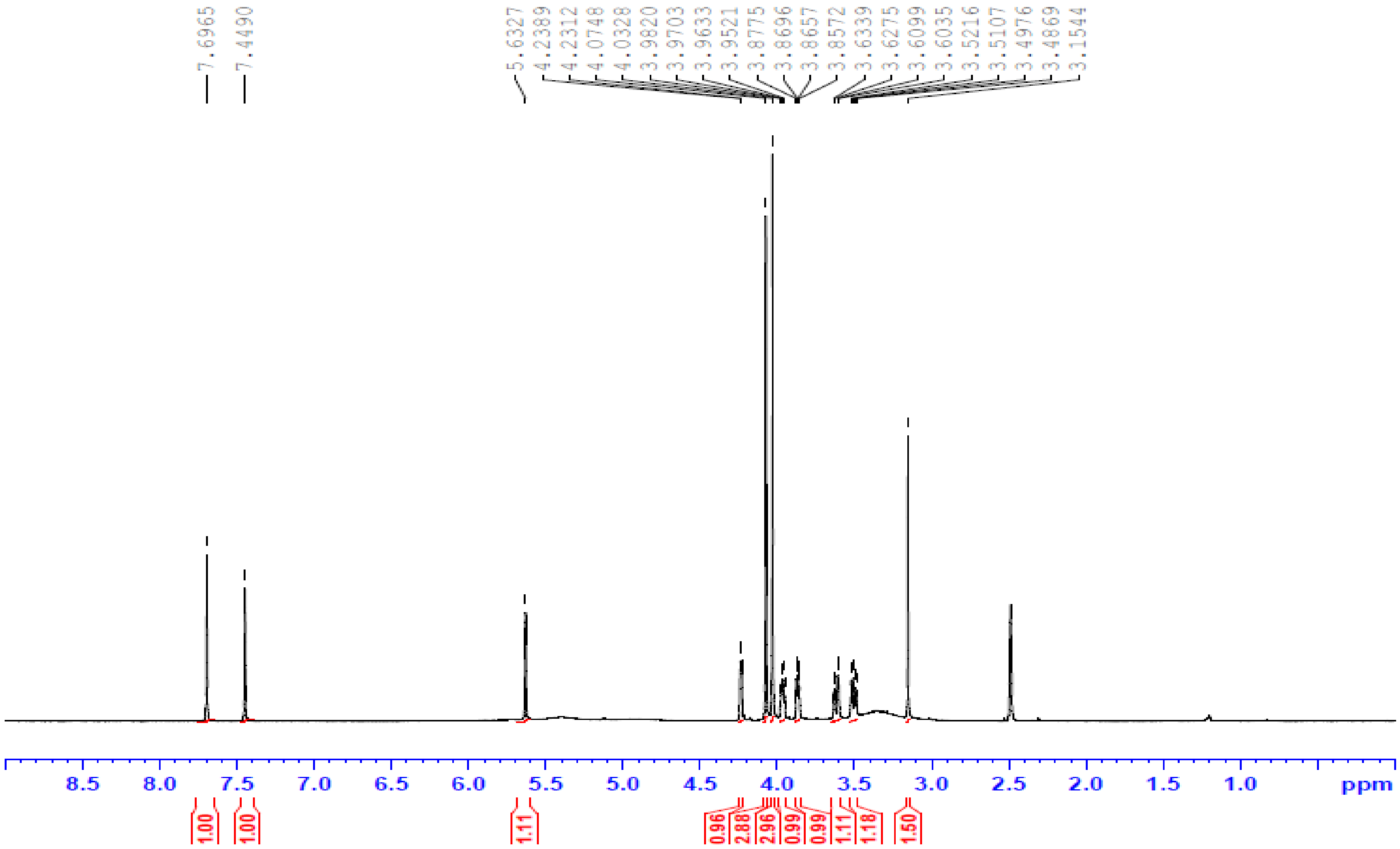

Figure S 1A. ^{1}H NMR (DMSO-d_6, 500 MHz) spectrum of **1**

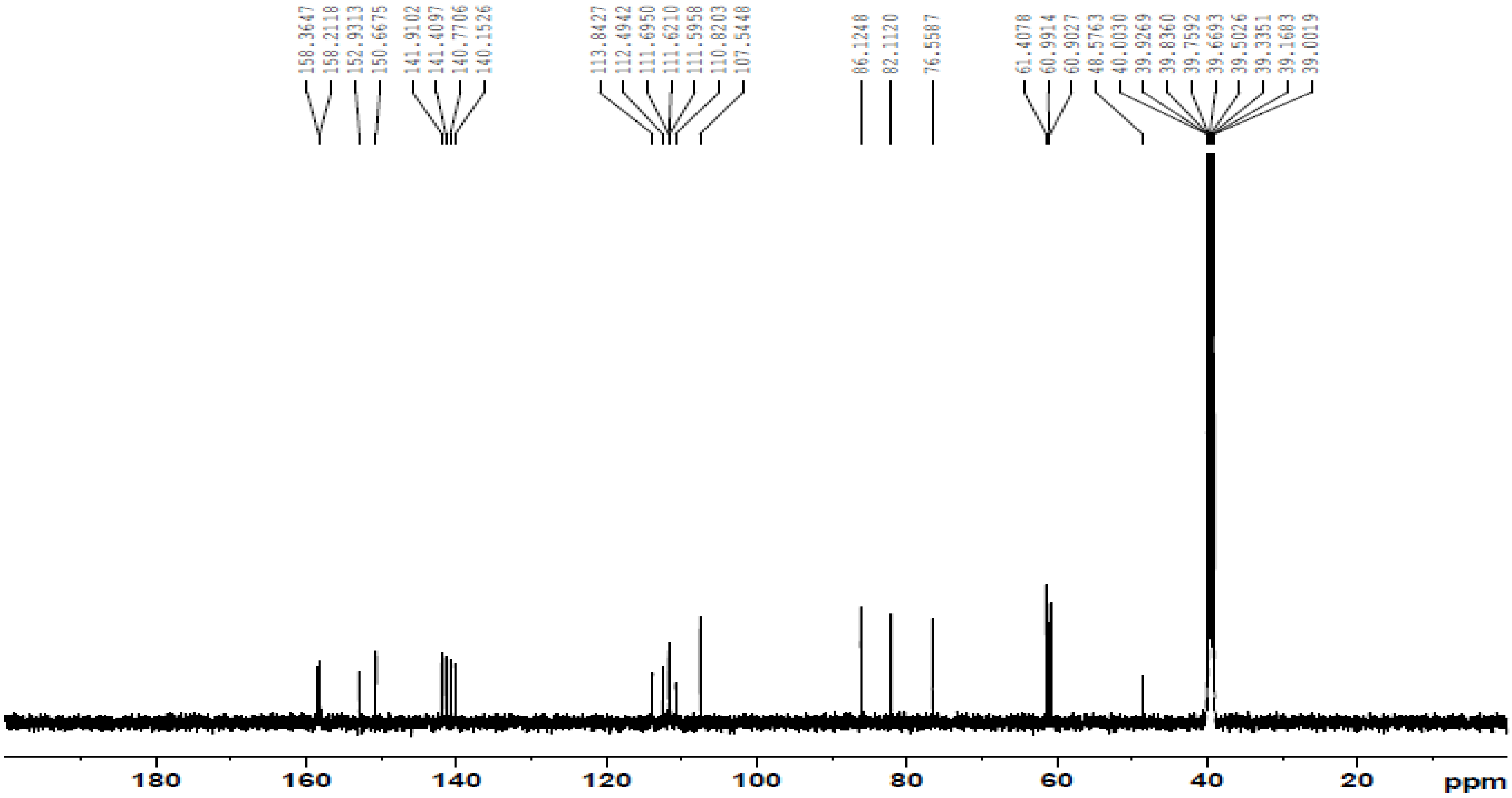

Figure S 1B. ^{13}C NMR (DMSO-d_6, 125 MHz) spectrum of **1**

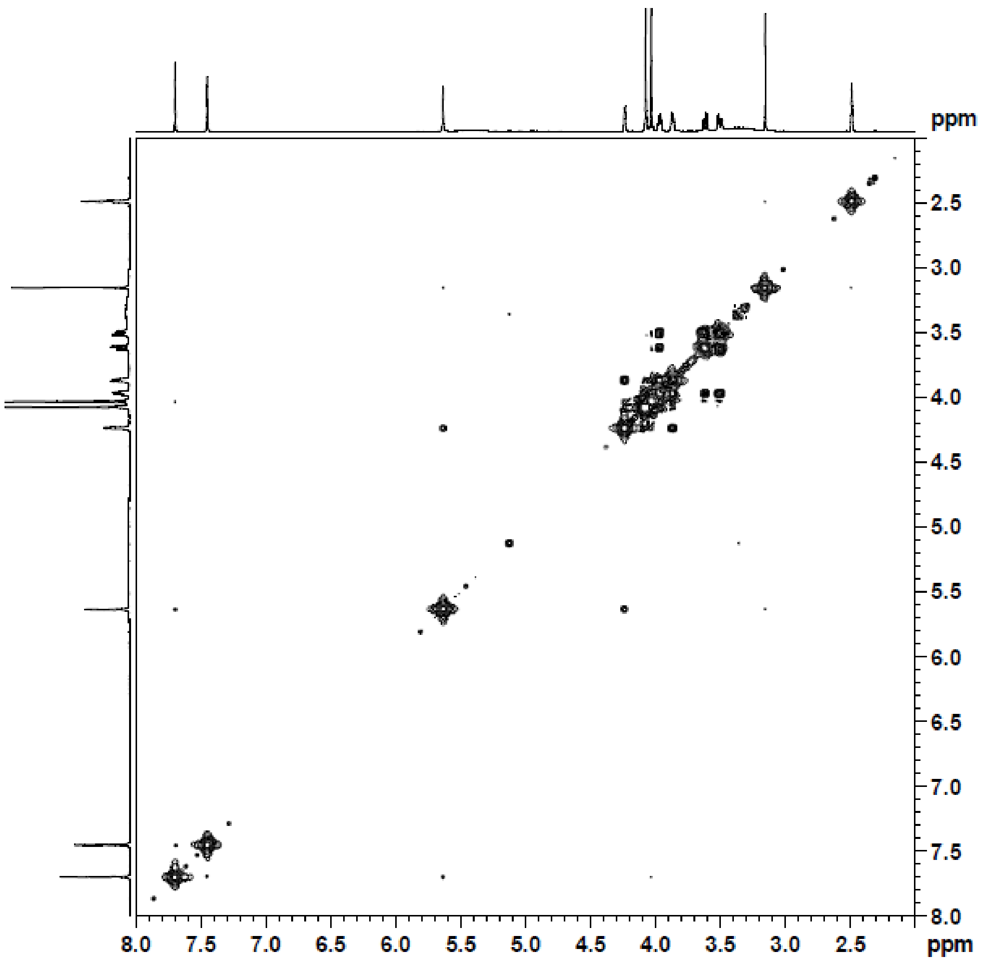

Figure S 1C. ^{1}H-^{1}H COSY spectrum of **1**

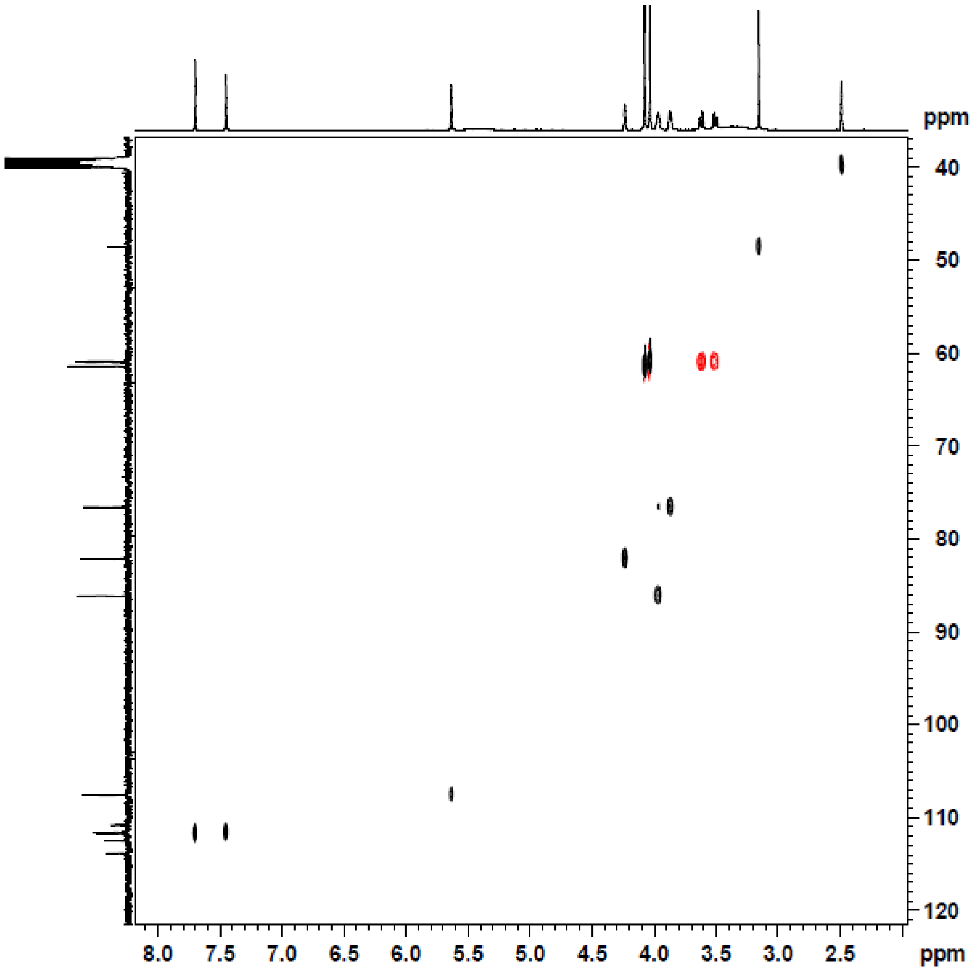

Figure S 1D. HSQC spectrum of **1**

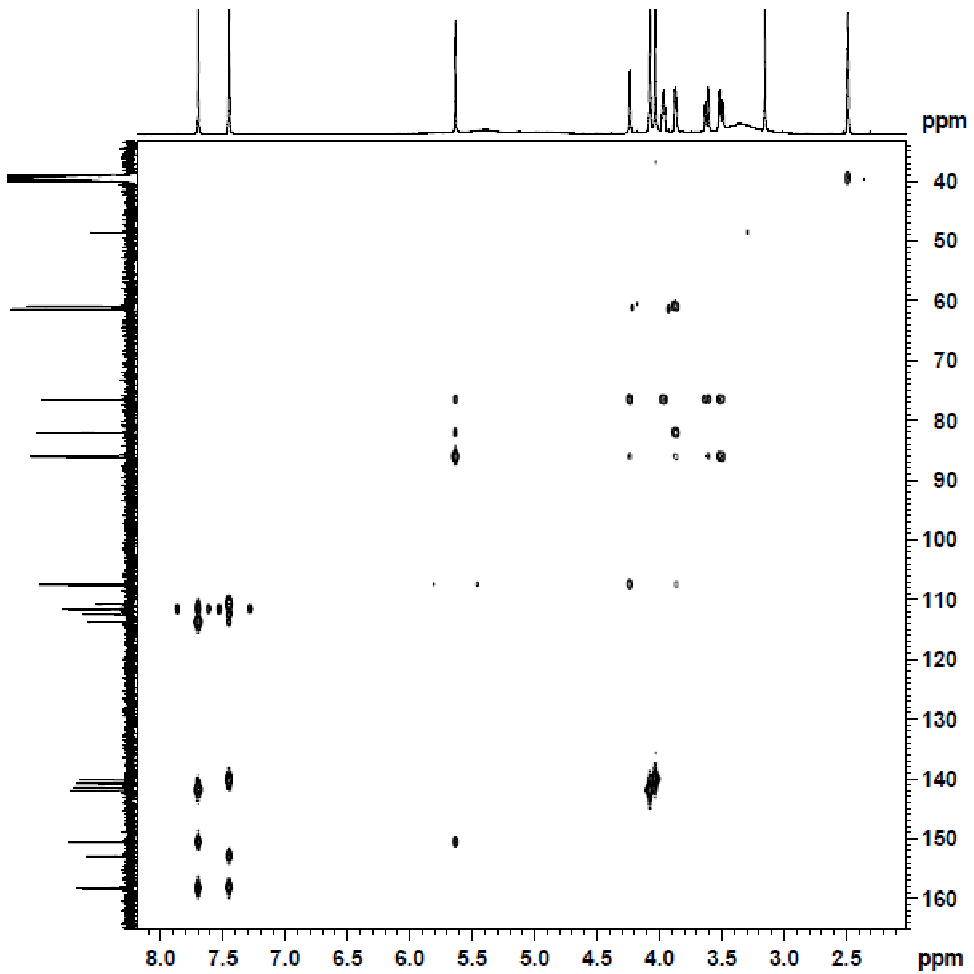

Figure S 1E. HMBC spectrum of **1**

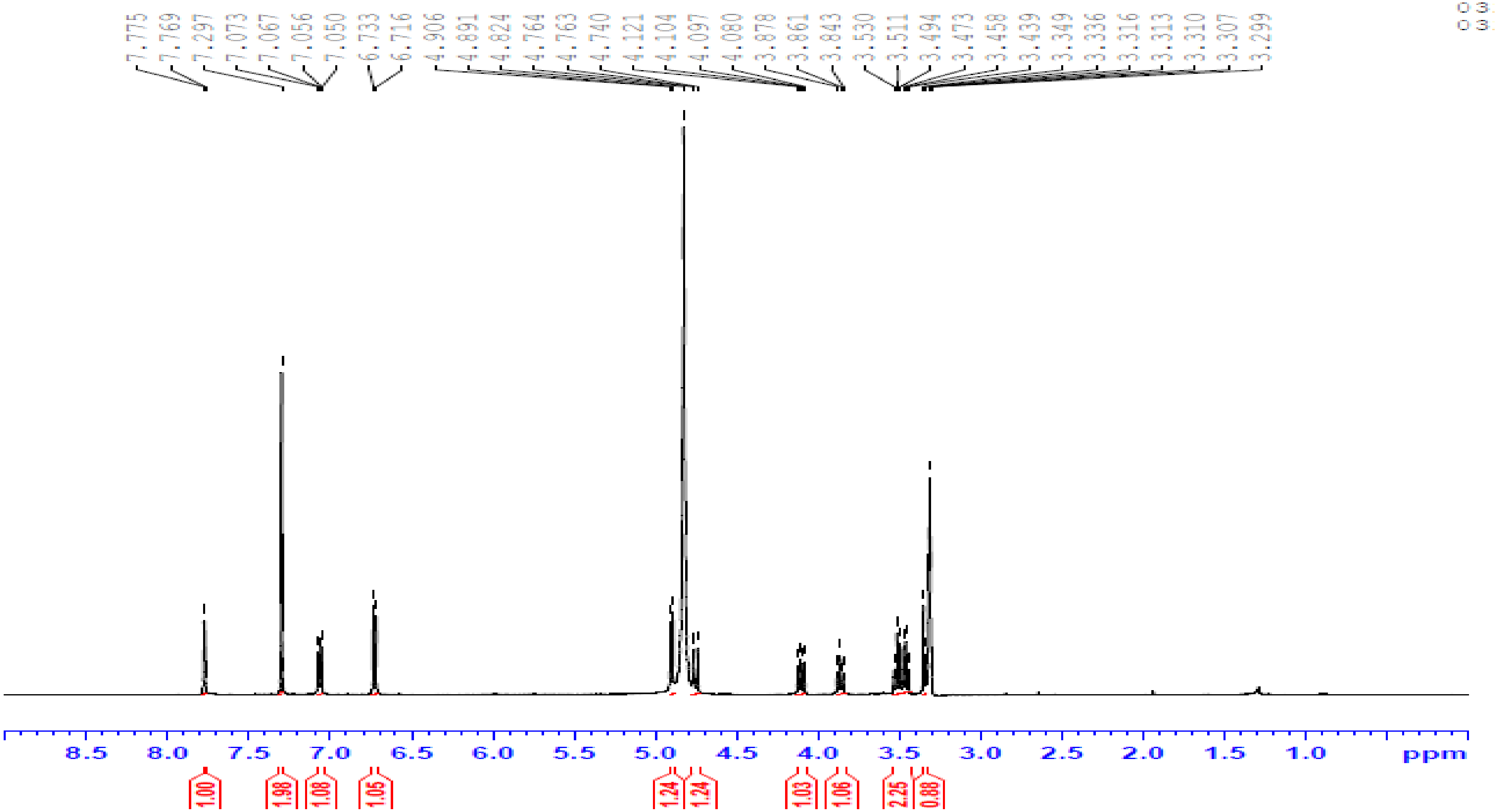

Figure S 2A. ^{1}H NMR (Methanol-d_4, 500 MHz) spectrum of **2**

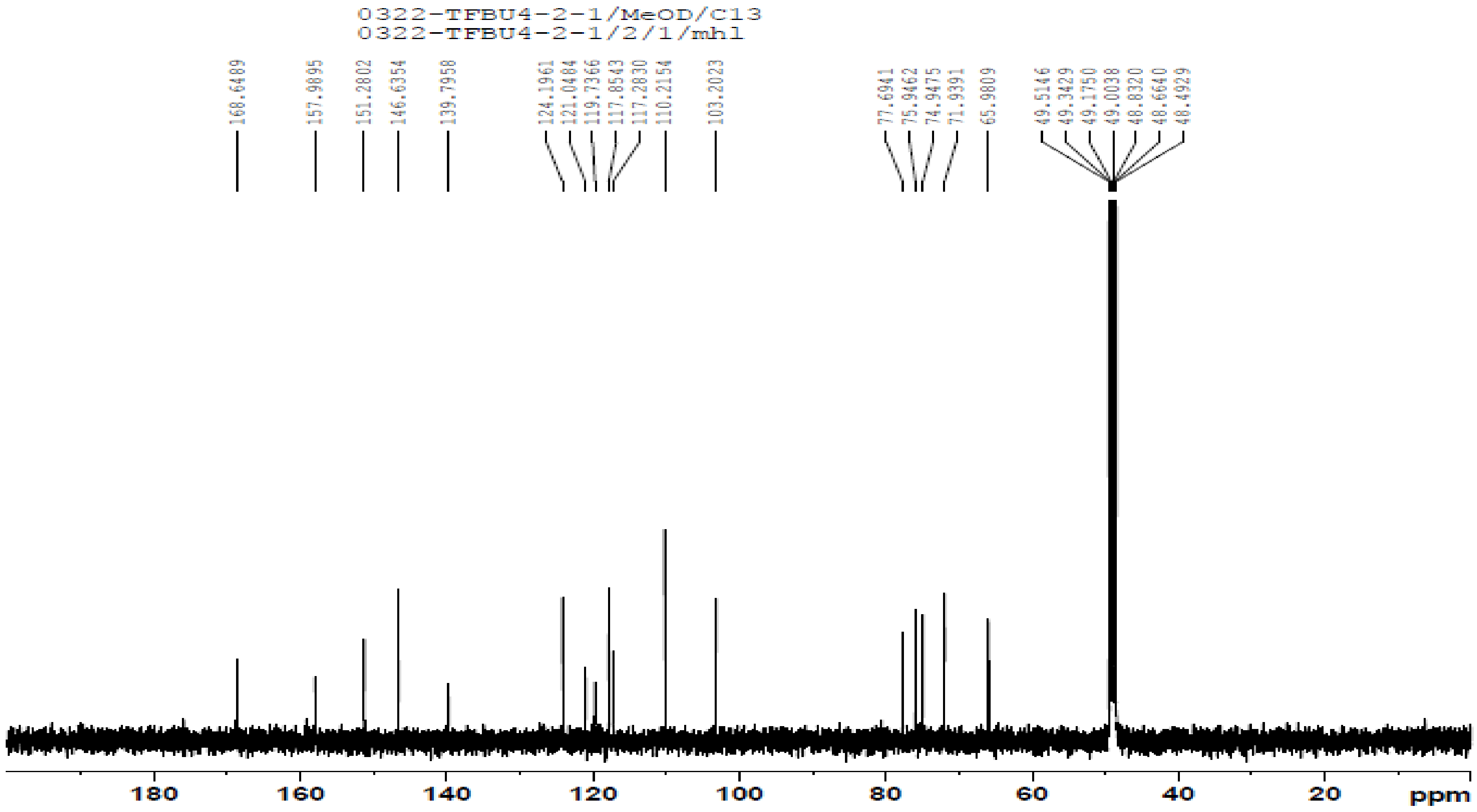

Figure S 2B. ^{13}C NMR (Methanol-d_4, 125 MHz) spectrum of **2**

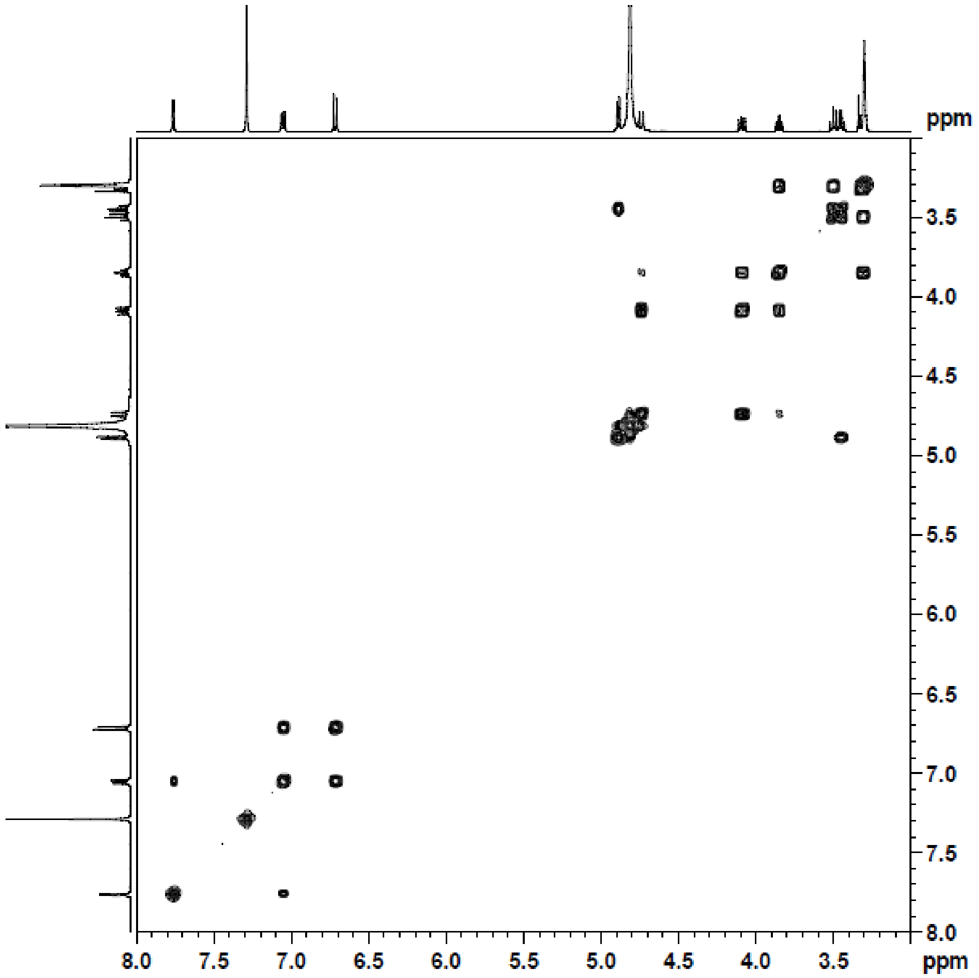

Figure S 2C. ^{1}H-^{1}H COSY spectrum of **2**

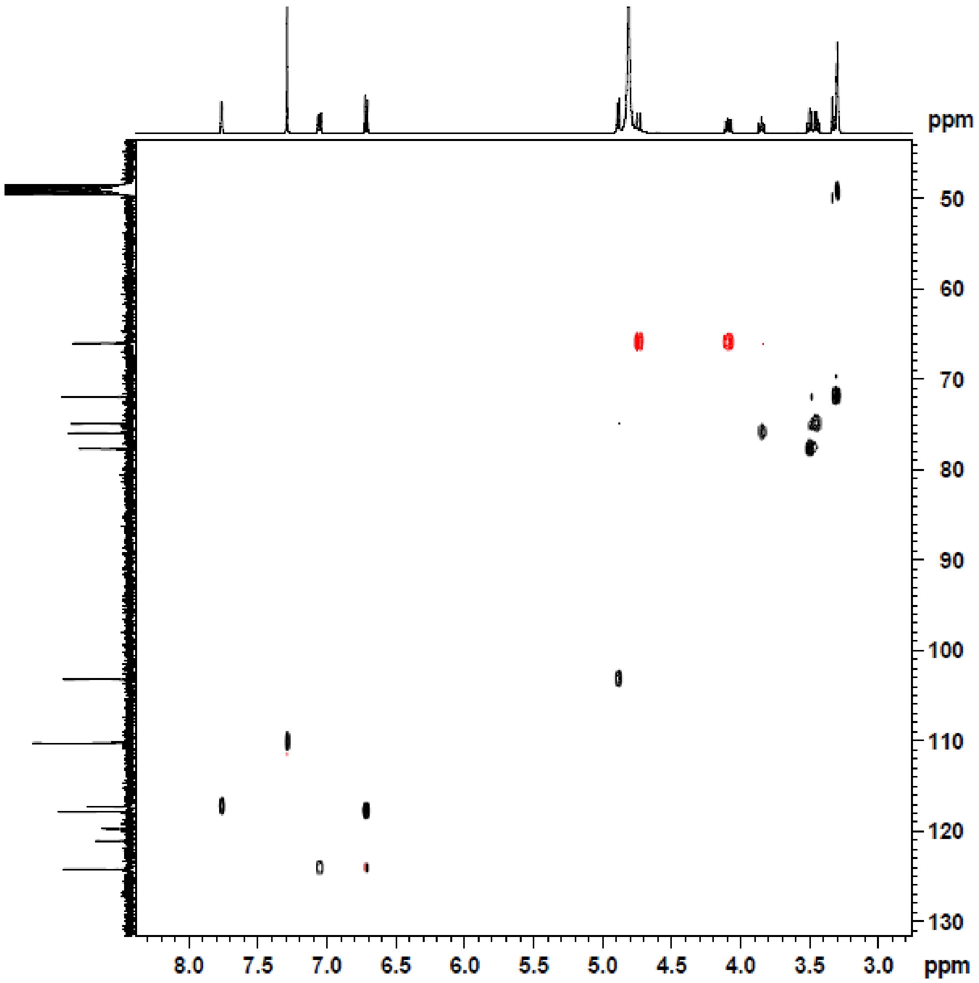

Figure S 2D. HSQC spectrum of **2**

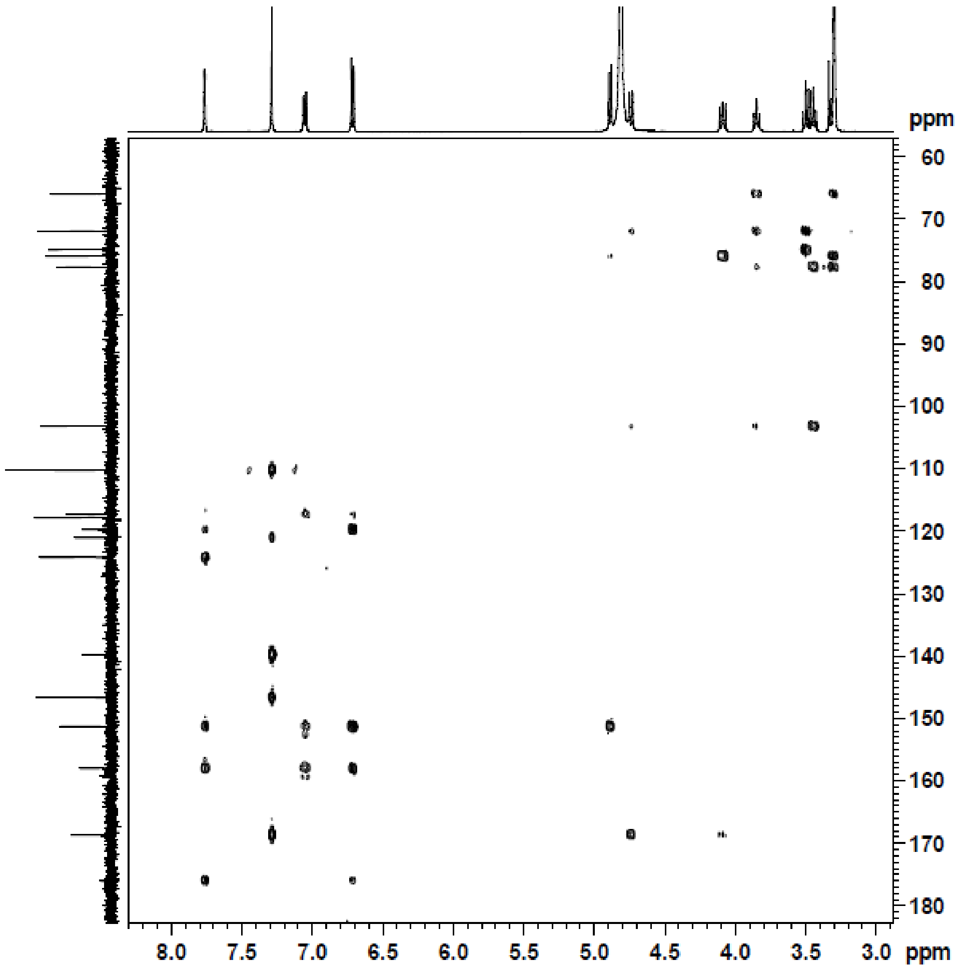

Figure S 2E. HMBC spectrum of **2**

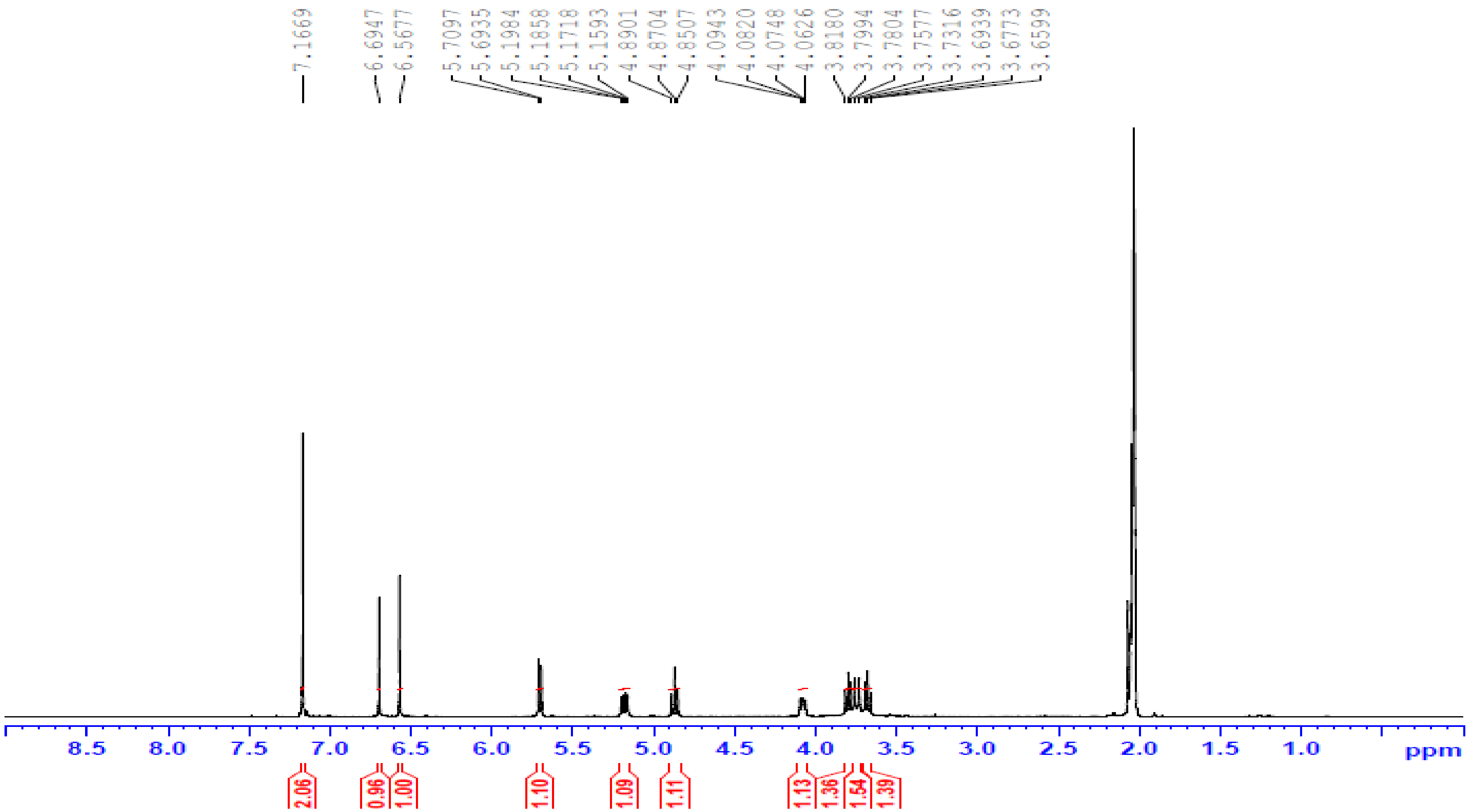

Figure S 3A. ^{1}H NMR (acetone-d_6, 500 MHz) spectrum of **3**

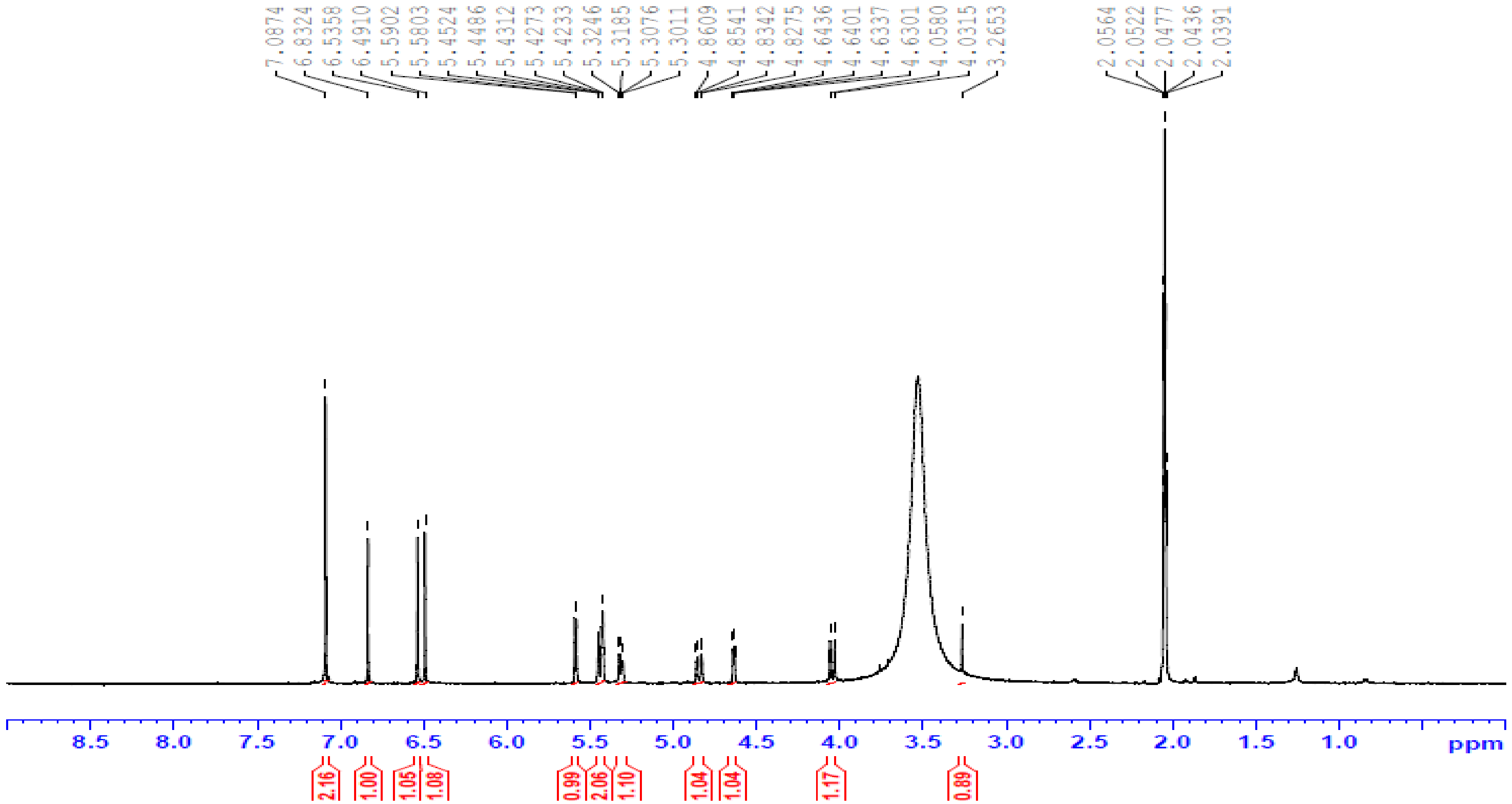

Figure S 4A. ^{1}H NMR (acetone-d_6, 500 MHz) spectrum of **4**

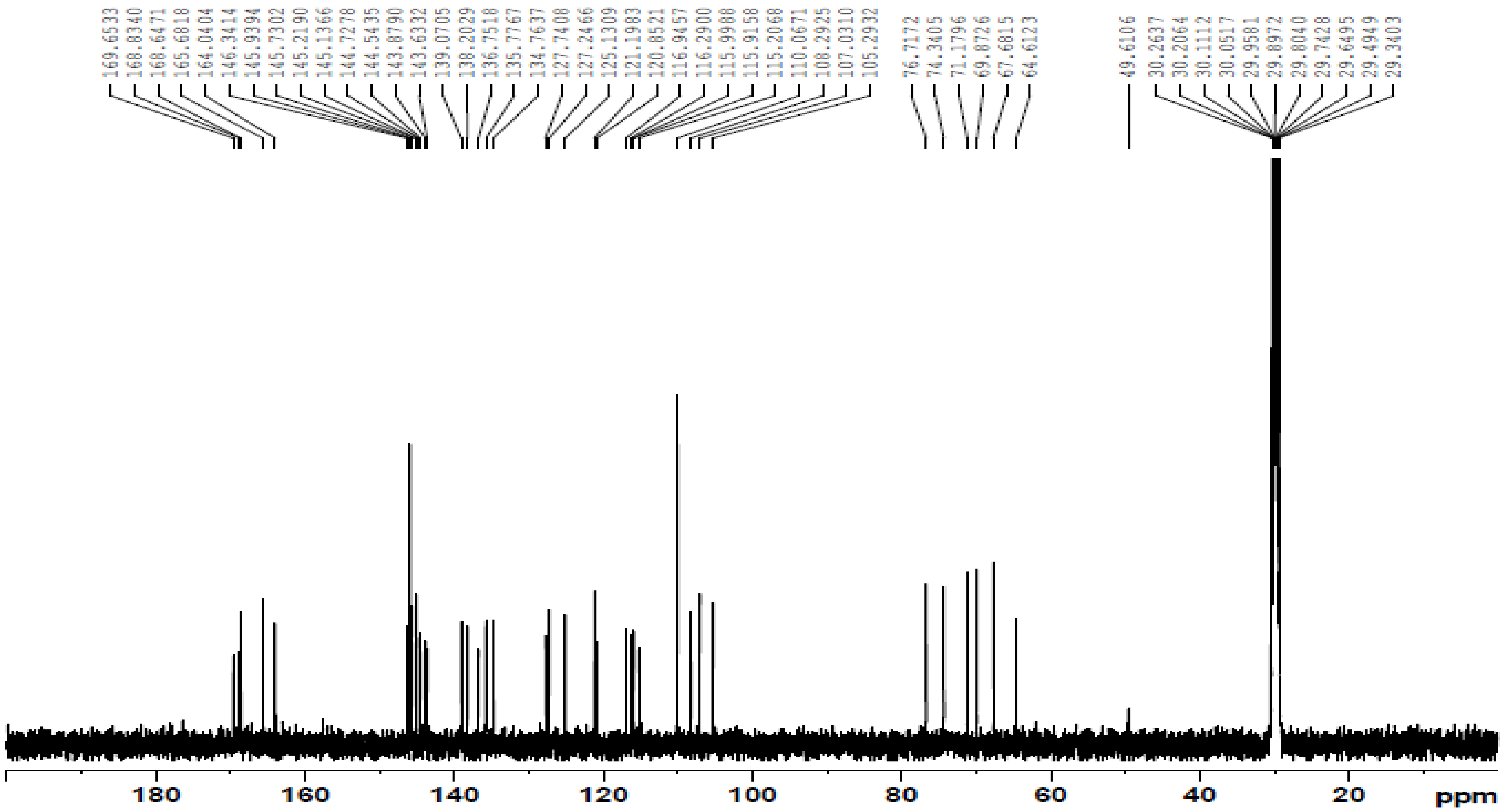

Figure S 4B. ^{13}C NMR (acetone-d_6, 125 MHz) spectrum of **4**

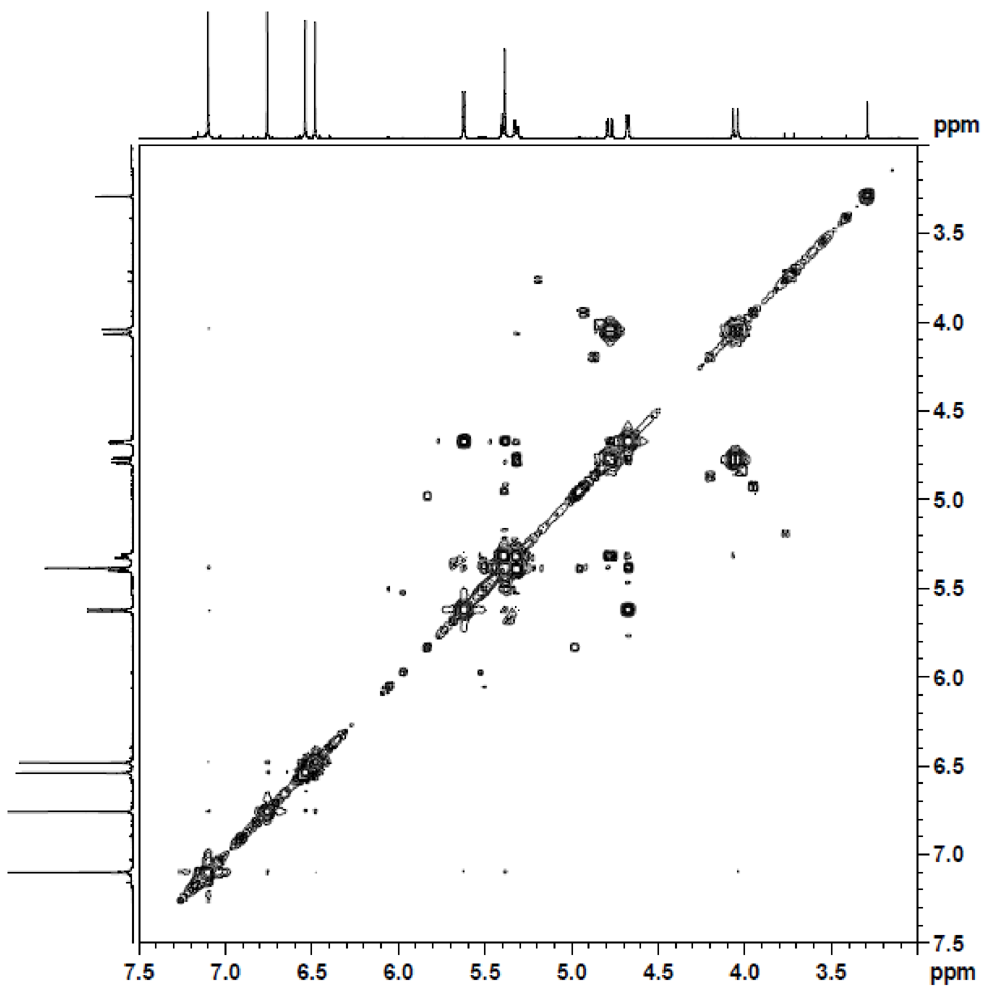

Figure S 4C. ^{1}H-^{1}H COSY spectrum of **4**

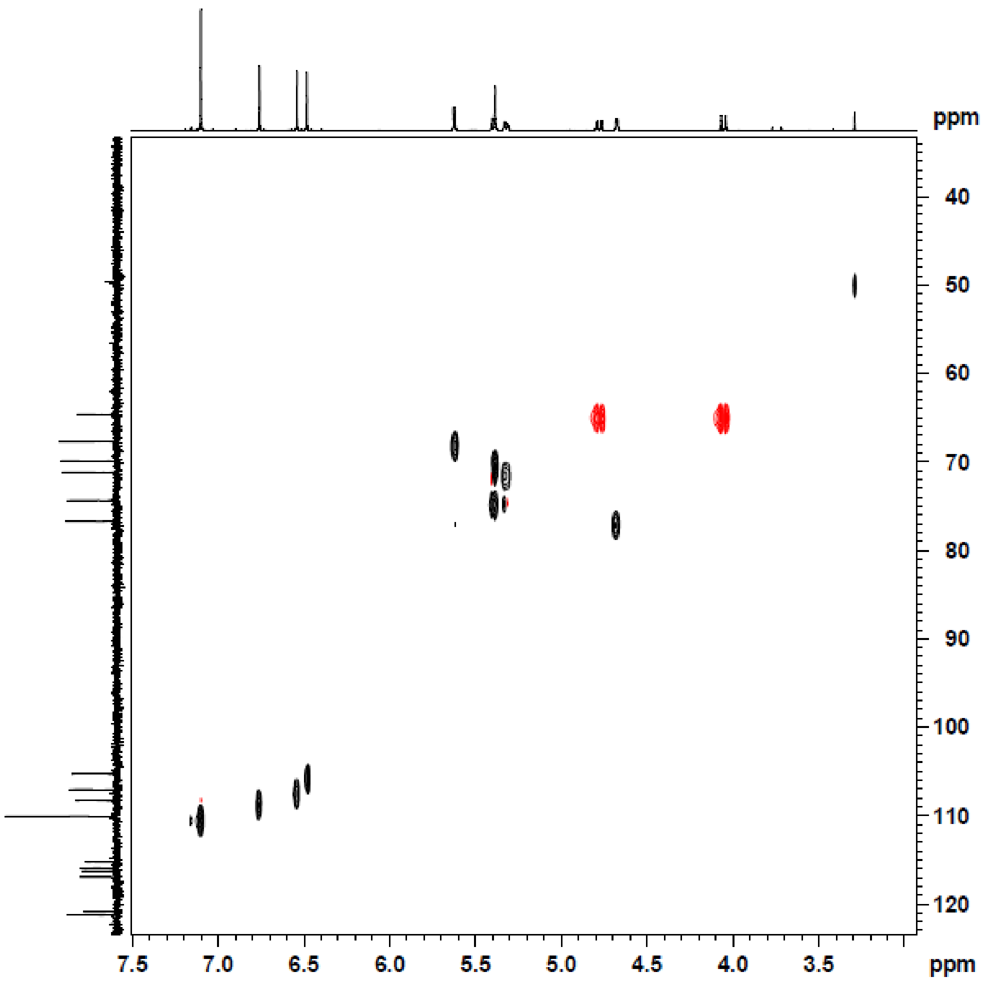

Figure S 4D. HSQC spectrum of **4**

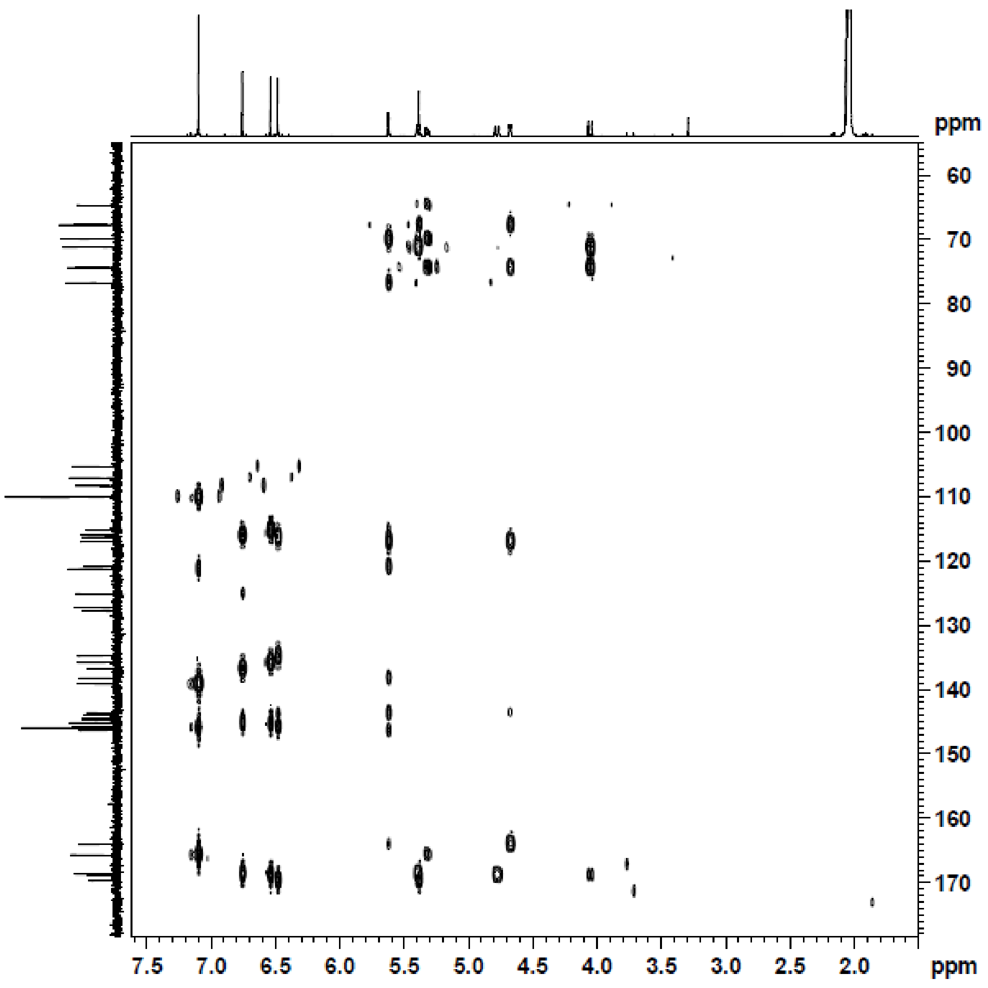

Figure S 4E. HMBC spectrum of **4**

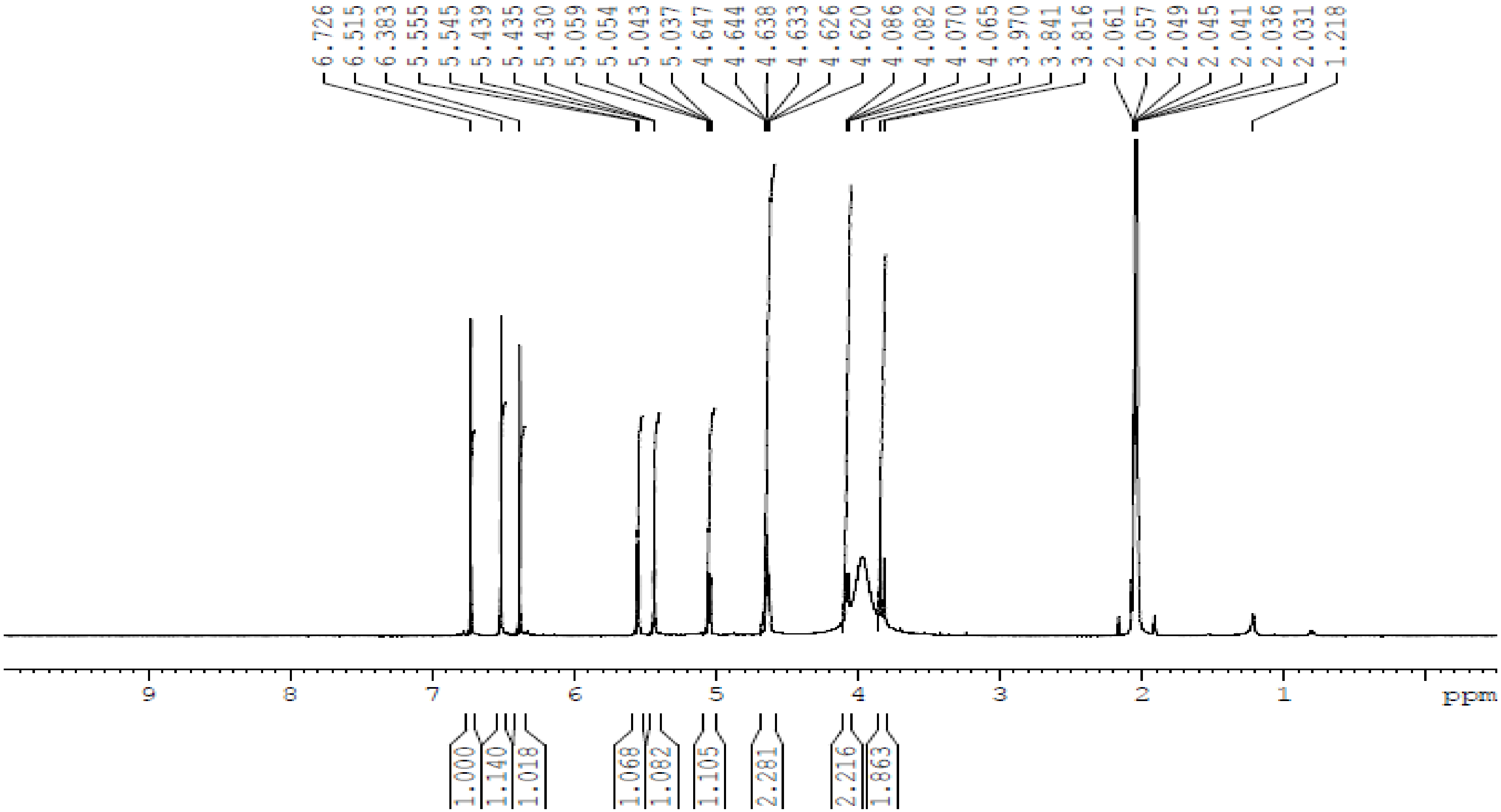

Figure S 5A. ^{1}H NMR (acetone-d_6, 500 MHz) spectrum of **5**

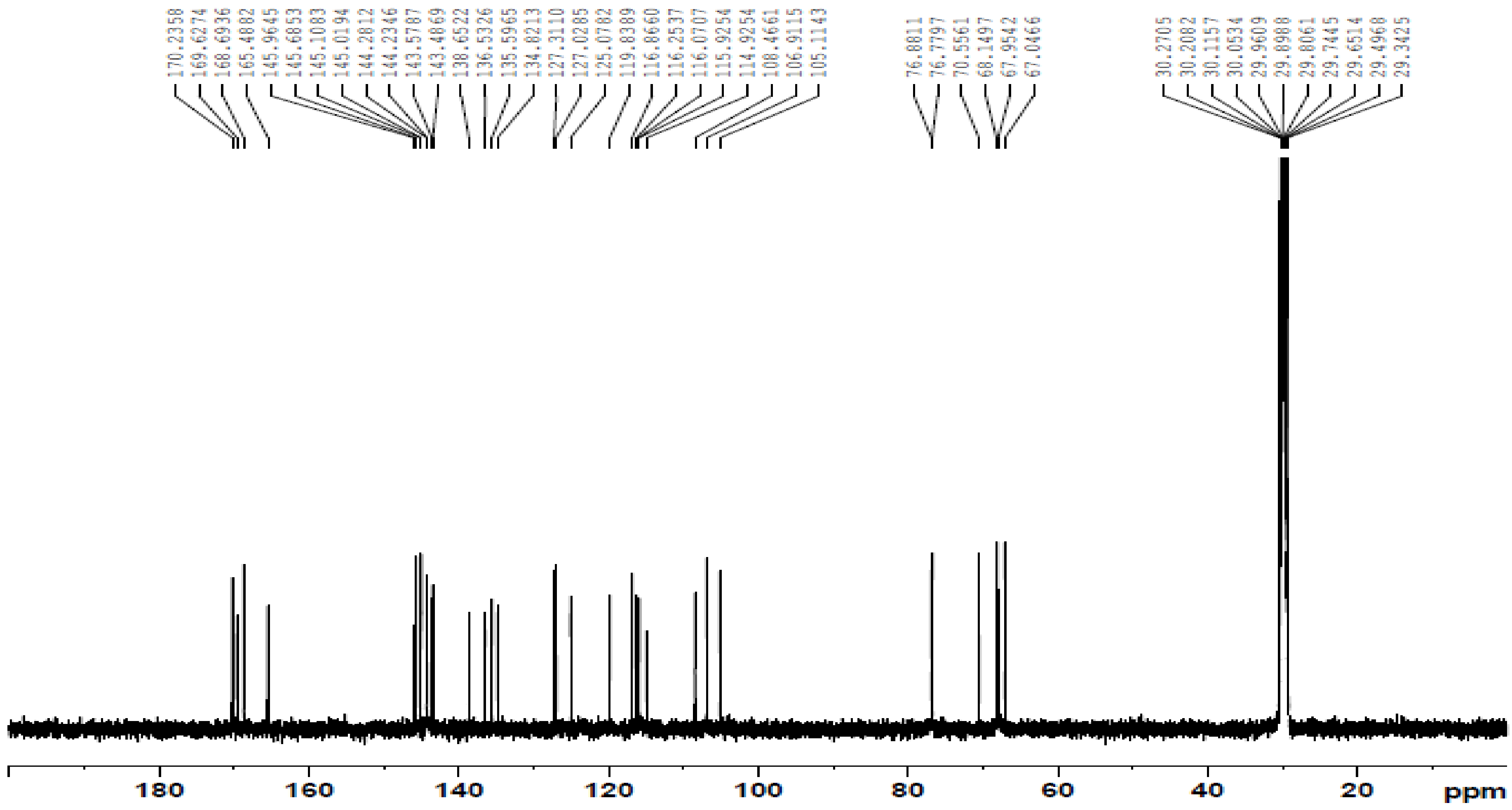

Figure S 5B. ^{13}C NMR (acetone-d_6, 125 MHz) spectrum of compound **5**

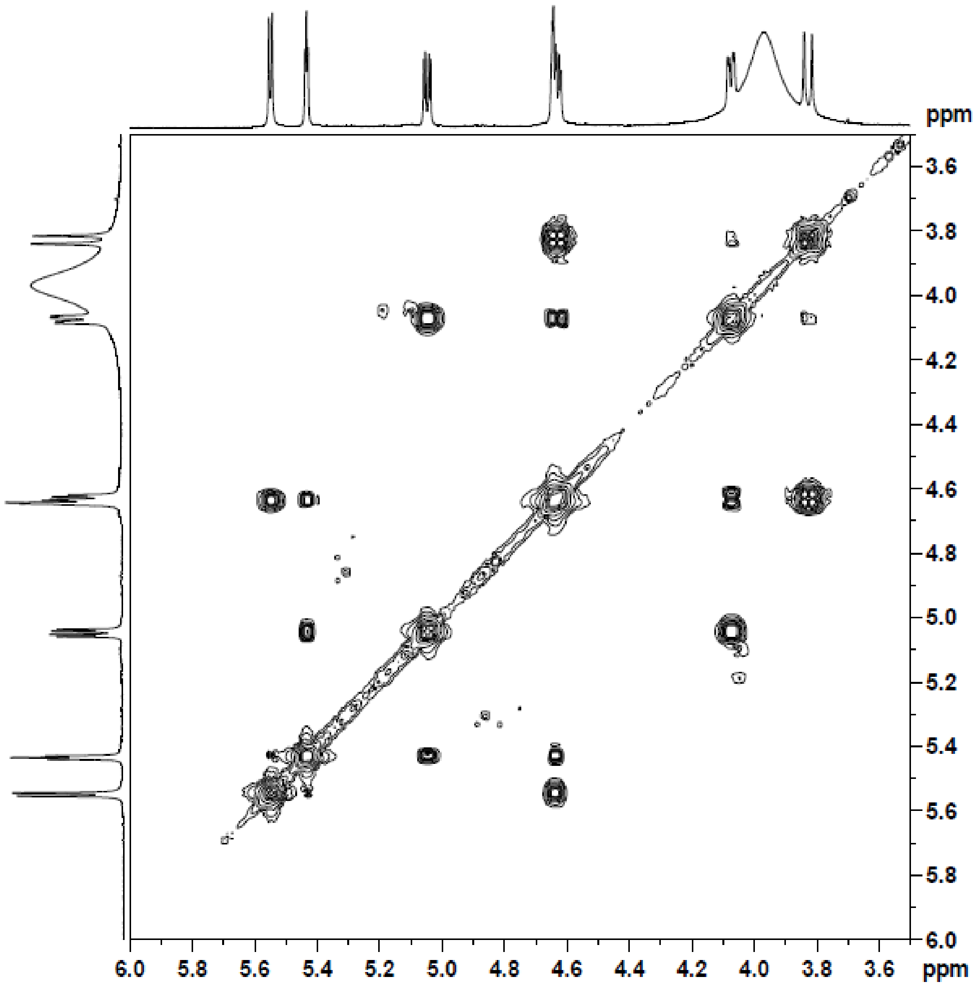

Figure S 5C. ^{1}H-^{1}H COSY spectrum of **5**

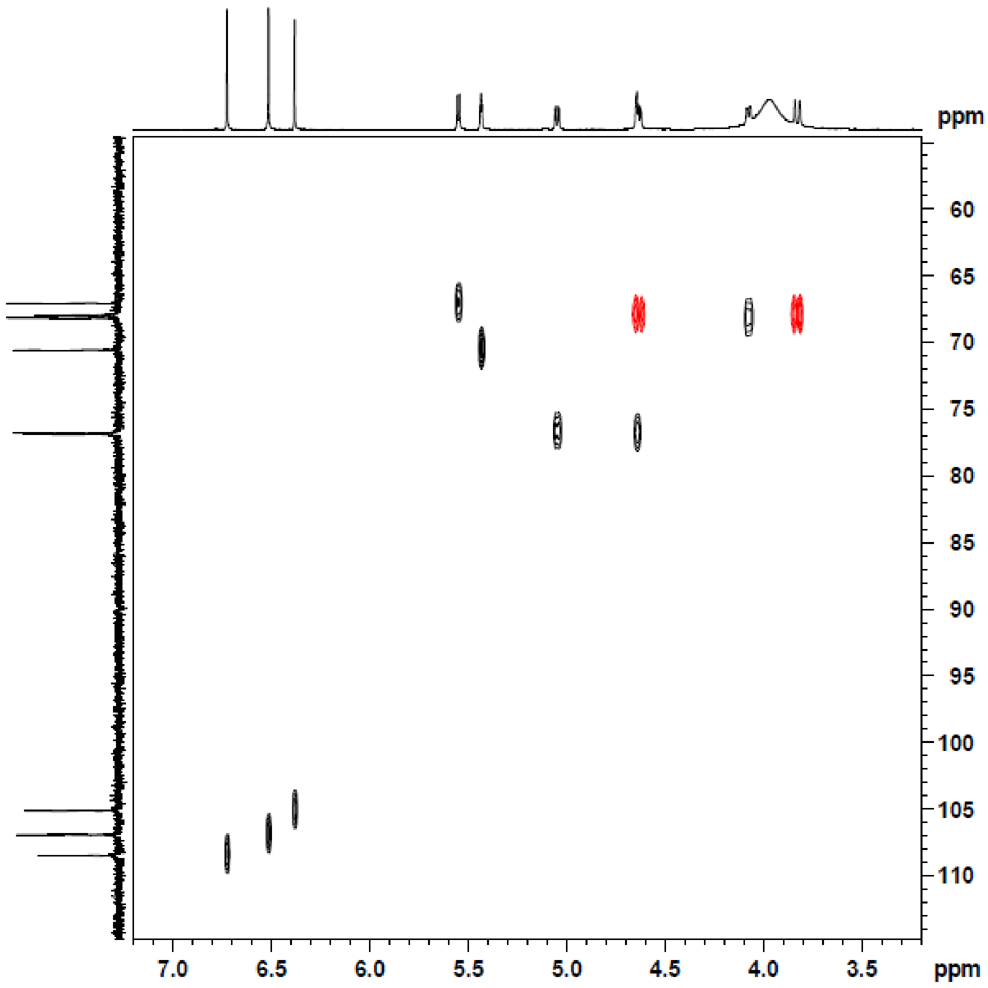

Figure S 5D. HSQC spectrum of **5**

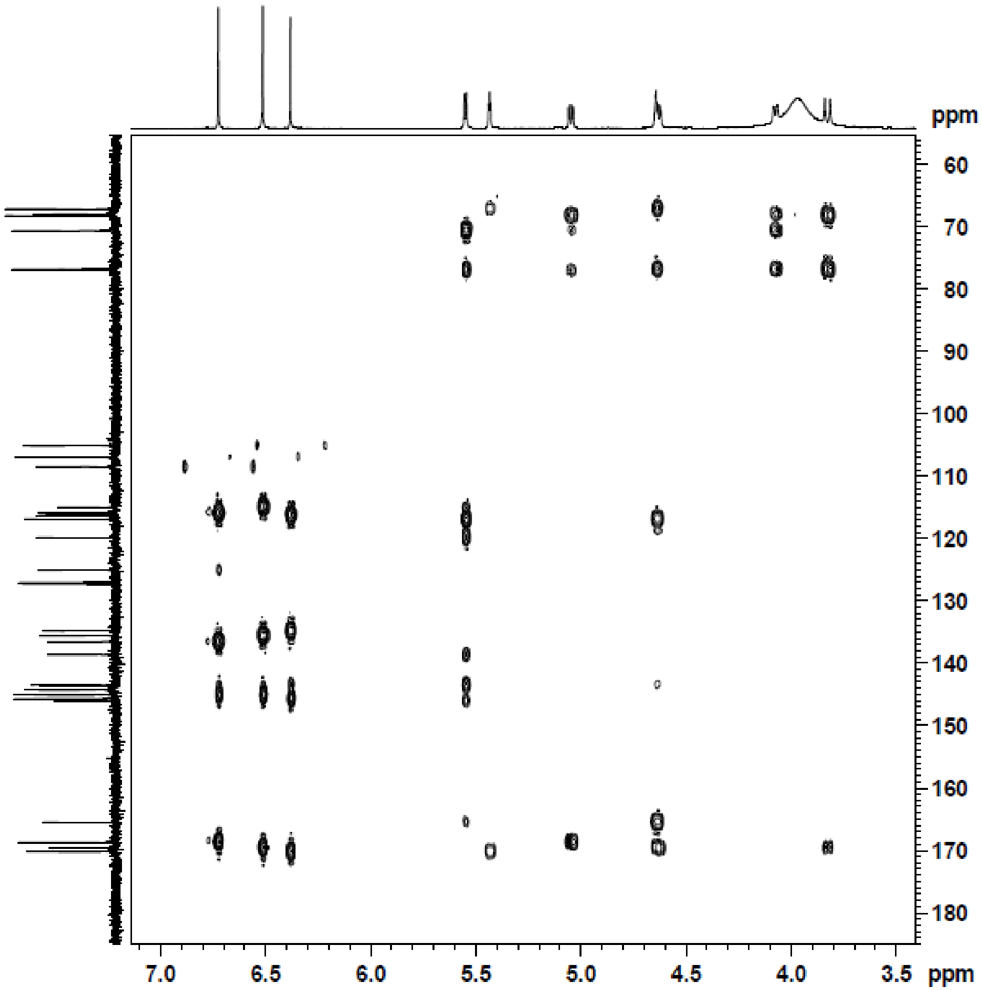

Figure S 5E. HMBC spectrum of **5**

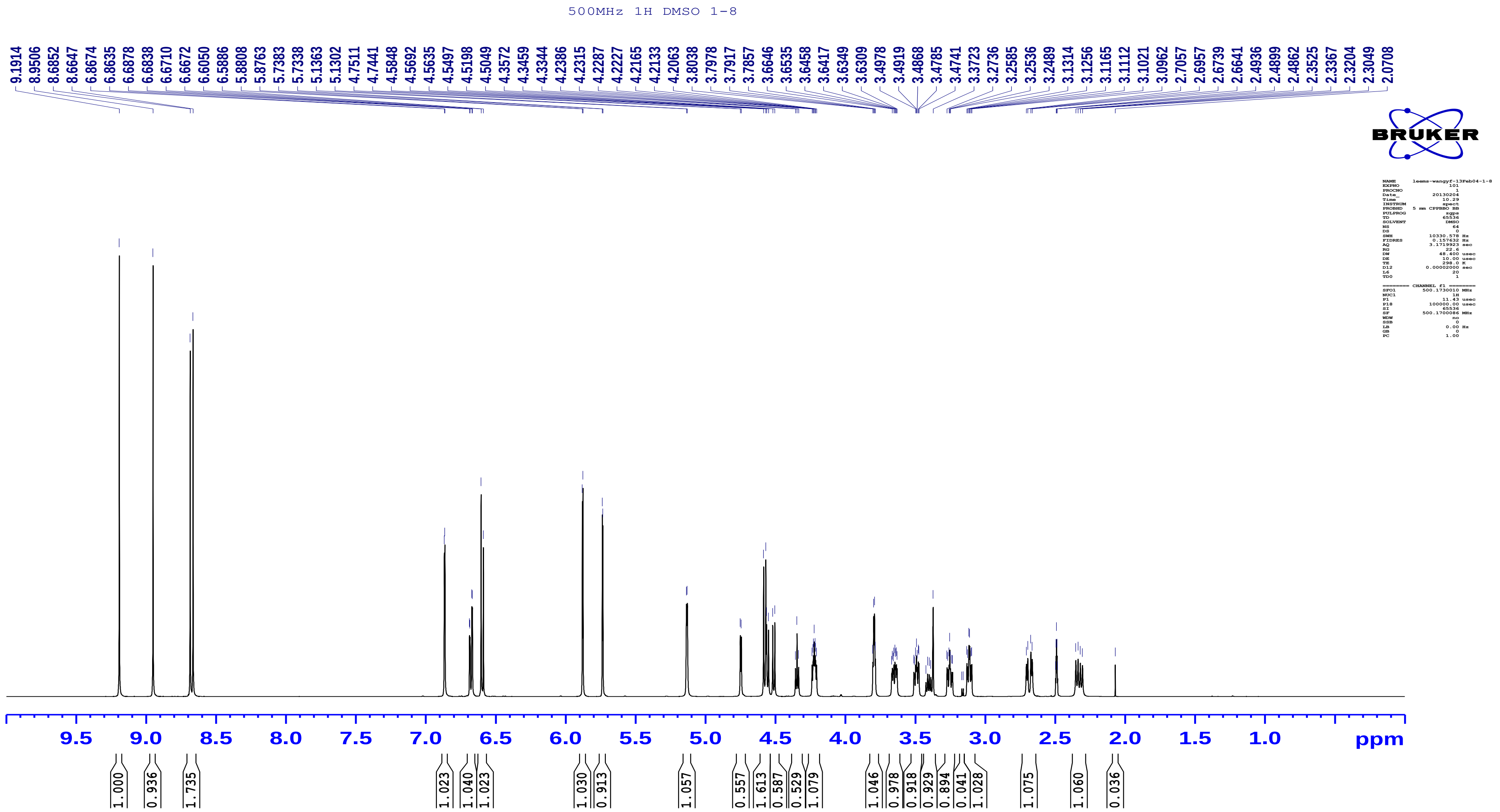

Figure S 6A. ^{1}H NMR (DMSO-d_6, 500 MHz) spectrum of **6**

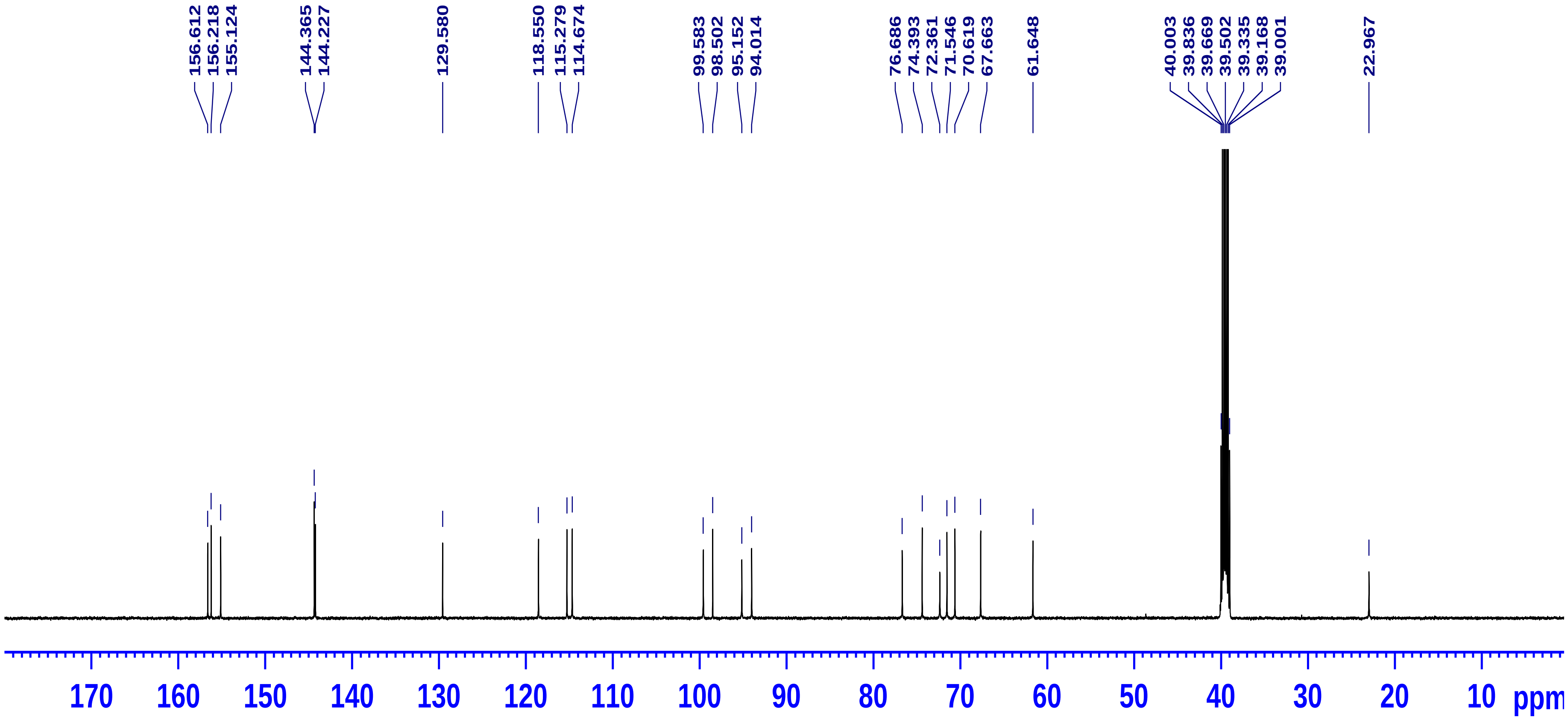

Figure S 6B. ^{13}C NMR (DMSO-d_6, 125 MHz) spectrum of **6**

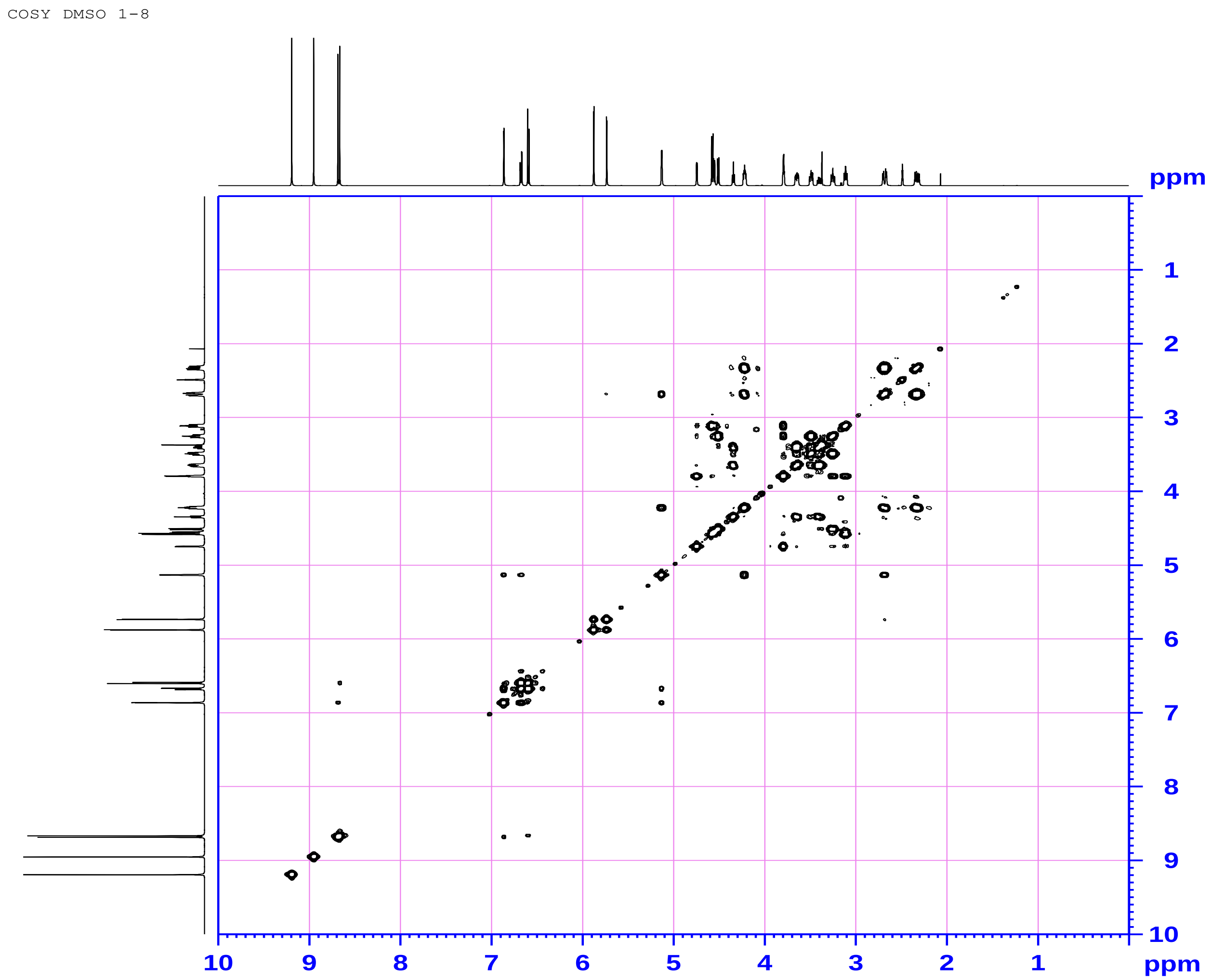

Figure S 6C. ^{1}H-^{1}H COSY spectrum of **6**

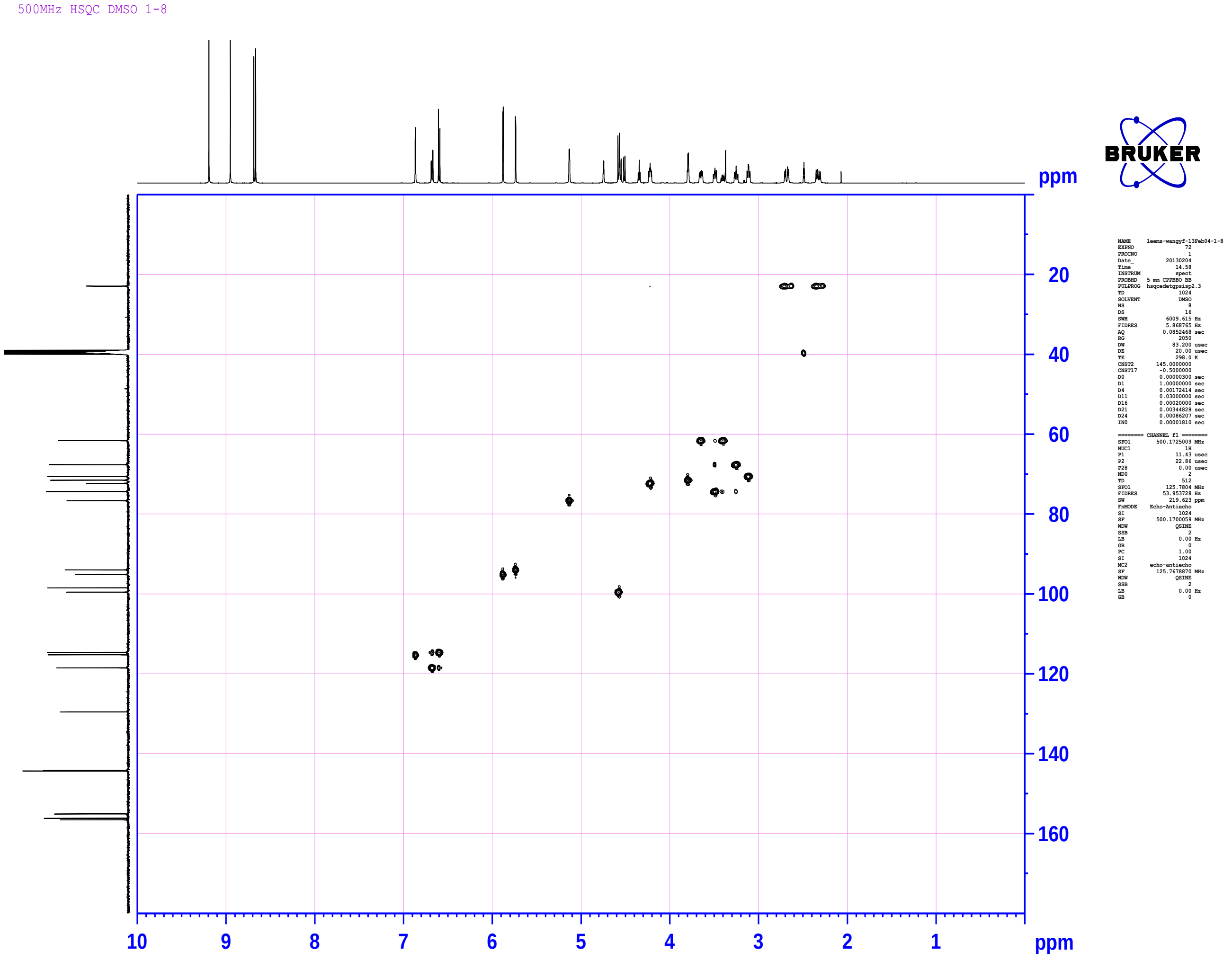

Figure S 6D. HSQC spectrum of **6**

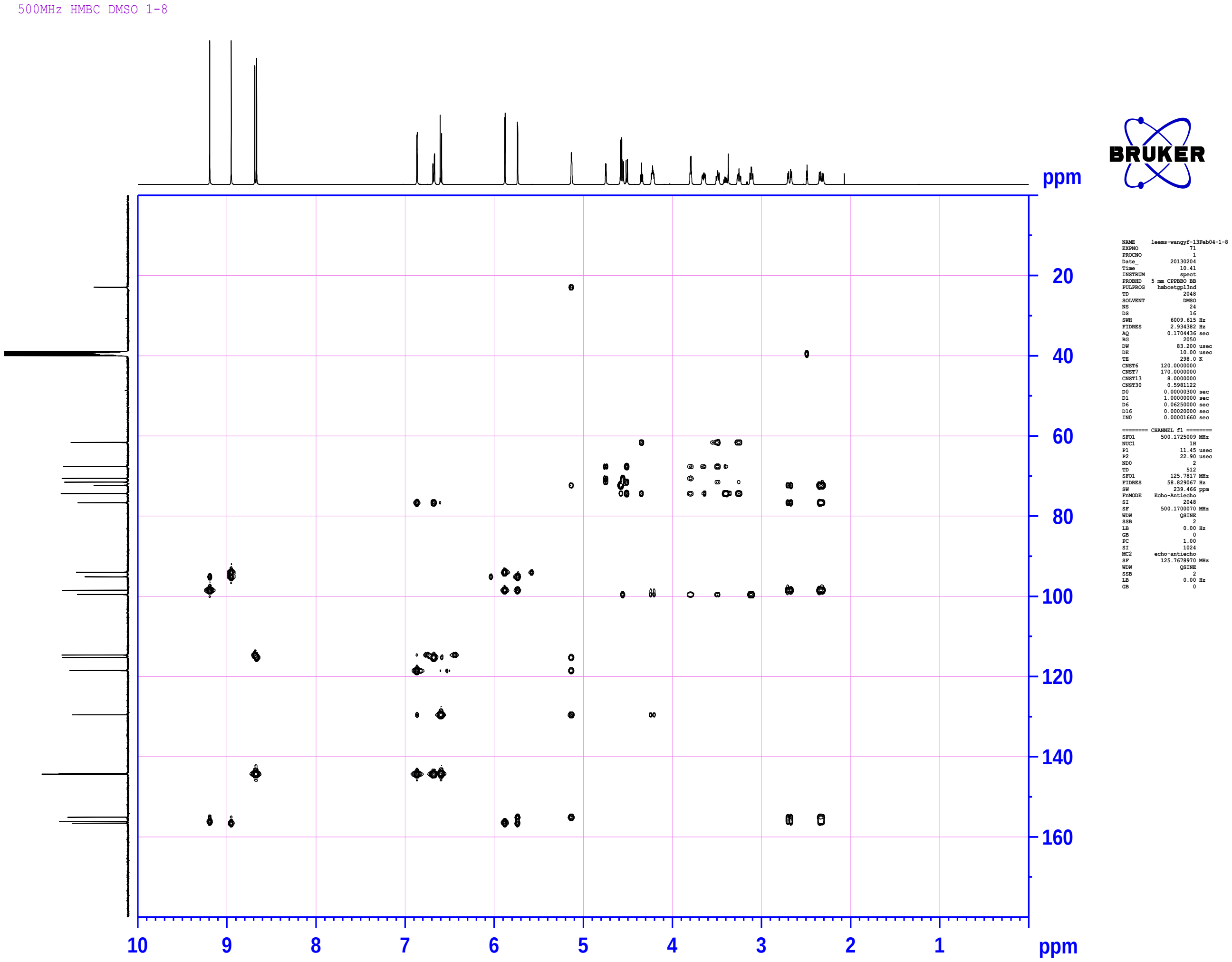

Figure S 6E. HMBC spectrum of **6**
